# Synthesis and Rational Design of New Appended 1,2,3-Triazole-uracil Ensembles as Promising Anti-Tumor Agents via In Silico VEGFR-2 Transferase Inhibition

**DOI:** 10.3390/molecules26071952

**Published:** 2021-03-30

**Authors:** Nadipolla Naresh Reddy, Sung-Jen Hung, Merugu Kumara Swamy, Ananthula Sanjeev, Vankadari Srinivasa Rao, Rondla Rohini, Atcha Krishnam Raju, Kuthati Bhaskar, Anren Hu, Puchakayala Muralidhar Reddy

**Affiliations:** 1 Department of Chemistry, Osmania University, Hyderabad, Telangana 500007, India; nareshnadipolla@gmail.com (N.N.R.); kumaraswamy.sci@gmail.com (M.K.S.); sanjeev.ku610@gmail.com (A.S.); chemsrinu44@gmail.com (V.S.R.); prmnreddy@osmania.ac.in (R.R.); kuthati18@osmania.ac.in (K.B.); 2 Department of Dermatology, Buddhist Tzu-Chi General Hospital, Hualien 97002, Taiwan; md.hong@msa.hinet.net; 3 Institute of Medical Sciences, Tzu-Chi University, Hualien 97002, Taiwan; 4 Department of Chemistry, Nizam College, Osmania University, Hyderabad 500001, India; krishnamrajua@osmania.ac.in; 5 Department of Laboratory Medicine and Biotechnology, College of Medicine, Tzu-Chi University, Hualien 97004, Taiwan

**Keywords:** drug design, 1,2,3-triazole-uracil, VEGFR-2, in silico docking, MTT assay, anti-cancer agents

## Abstract

Angiogenesis inhibition is a key step towards the designing of new chemotherapeutic agents. In a view to preparing new molecular entities for cancer treatment, eighteen 1,2,3-triazole-uracil ensembles **5a**–**r** were designed and synthesized via the click reaction. The ligands were well characterized using ^1^H-, ^13^C-NMR, elemental analysis and ESI-mass spectrometry. The in silico binding propinquities of the ligands were studied sequentially in the active region of VEGFR-2 using the Molegro virtual docker. All the compounds produced remarkable interactions and potentially inhibitory ligands against VEGFR-2 were obtained with high negative binding energies. Drug-likeness was assessed from the ADME properties. Cytotoxicity of the test compounds was measured against HeLa and HUH-7 tumor cells and NIH/3T3 normal cells by MTT assay. Compound **5h** showed higher growth inhibition activity than the positive control, 5-fluorouracil (5-FU), against both HeLa and HUH-7 cells with IC_50_ values of 4.5 and 7.7 μM respectively. Interestingly, the compounds **5a**–**r** did not show any cytotoxicity towards the normal cell lines. The results advance the position of substituted triazoles in the area of drug design with no ambiguity.

## 1. Introduction

Cancer is a disease with a high mortality rate that accounts for more than 9 million deaths every year [1,2]. The discovery of molecules to treat cancer with minimal or no side effects is of great importance in medicinal chemistry. To date, over 100 anti-cancer agents are approved for chemotherapy [3]. Due to the increasing resistance to existing drugs, there is a need for the synthesis of new agents which can effectively treat various cancers. The present investigation attempts to develop new therapeutic agents assembled using 1,2,3-triazoles and uracil moieties. 1,2,3-Triazoles contain three nitrogens in the ring system and are an important class of heterocyclic compounds with remarkable biological properties [4]. It is known that they possess promising antiviral, antifungal and anticancer, antileishmanial, antituberculosis, anti-inflammatory, antimicrobial and antibacterial activity [5,6,7,8]. It is assumed that the presence of heteroatoms directly affects their toxicity towards cancer cells [9]. Out of many synthetic routes for the preparation of 1,2,3 triazoles, the Hüisgens thermal 1,3 dipolar cycloaddition is the classical and traditional approach that involves the interaction of terminal alkynes with organic azides [10]. The reaction proceeds via a concerted mechanism yielding 1,4- and 1,5-disubstituted triazoles. Cu(I) catalyzed reactions are reported to exclusively produce 1,4 disubstituted triazoles [10]. On the other hand, uracil derivatives were proved to display bactericidal, herbicidal and insecticidal properties [11]. Uracil moiety in combination with 1,2,3-triazoles recently proved to possess potent anticancer activities [12]. 5-Fluorouracil has long been known to inhibit tumor growth and was used as a standard drug in the current study [13].

Rational drug design is an important and widely accepted approach in the discovery of new drug candidates for various diseases. It includes the study of in silico docking interactions followed by an in vitro verification of the synthesized compounds. To account for the small molecule and target protein interactions, the Moldock program was utilized. Moldock is a high accuracy technique to predict protein-ligand interactions. It operates on a heuristic search algorithm that incorporates differential evolution along with a cavity prediction tool. The Moldock scoring function is an extension of the piecewise linear potential that includes new hydrogen-bonded and electrostatic terms [14]. The accuracy of Moldock was further improved by introducing a new rerank scoring function. It is established that the overall performance of the Moldock program is better than that of Flexx, Gold and Surflex [15].

**Figure 1:**
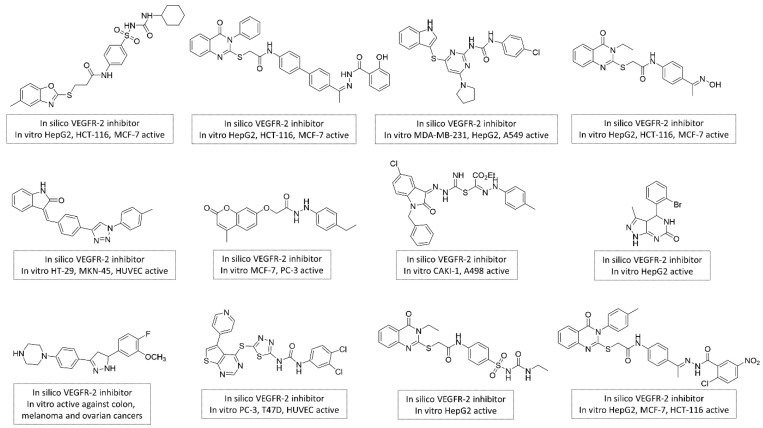
Recently reported drug candidates that proved to inhibit VEGFR-2 and emerged as effective molecules against various cancers.

Angiogenesis is a crucial physiological process that regulates the formation of new blood vessels [16]. This process is greatly exploited by genetically altered cancer cells for their proliferation, progression and metastasis [17,18]. The unwanted tumor cell proliferation can be blocked by employing suitable drug candidates that specifically hinder angiogenesis by interfering with the VEGFR signalling pathway. The vascular endothelial growth factor receptors (VEGFR1/VEGFR2/VEGFR3) are transmembrane proteins and only VEGFR-2 triggers the sprouting of new capillary blood vessels through VEGFR receptor signalling [19,20]. Disabling the functions of VEGFR-2 protein is a key step in drug discovery and it has become an undisputed target for new chemotherapeutic agents [21,22,23,24]. Recently, Heba et al., reported the docking interactions of 1,2-disubstituted benzimidazoles in the active site of VEGFR-2. The results depicted sharp hydrogen bond or hydrophobic interactions with the receptor protein and the ligands produced remarkable VEGFR-2 inhibition activity in HepG2 cell line with IC_50_ values as low as 1.98 μM [25]. Novel thiazolidine 2,4-diones reported by Khaled et al., have also demonstrated excellent in vitro VEGFR-2 inhibition and MCF-7 tumor growth suppression. Moreover, in silico VEGFR-2 binding results of synthesized ligands were very well correlated with in vitro antiproliferative activities [26]. Figure 1 depicts some recently developed in silico VEGFR-2 inhibitors that eventually evolved into potential in vitro anti-tumor agents [22,27,28,29,30,31,32,33,34,35,36,37]. These results justify the usage of VEGFR-2 protein in a rational drug design approach for the identification of new molecular agents for treating cancer.

Because of the synthesis accessibility, excellent biological properties of triazoles and uracils and VEGFR-2′s role in identifying potential anti-cancer agents, in this work we designed and isolated eighteen new 1,2,3-triazole-uracil scaffolds. The synthesized ligands were docked into the active site of VEGFR-2 receptor protein using the Molegro virtual docking program to estimate their inhibition properties. Finally, the compounds were screened for their anti-cancer activity against HeLa and HUH-7 tumor cell lines using standard MTT assay.

## 2. Results and Discussion

### 2.1. Chemistry

**Scheme 1:**
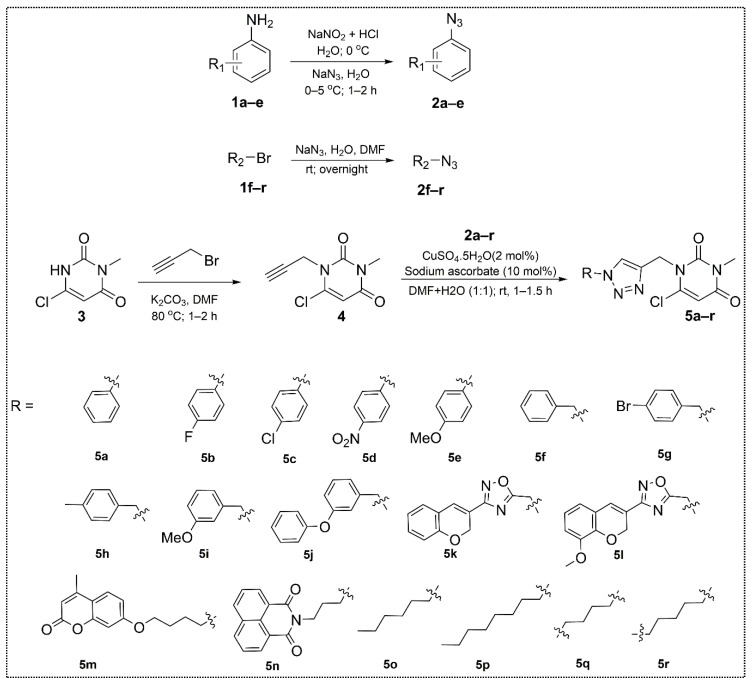
Synthetic route for the preparation of new 1,2,3-triazole-uracil scaffolds **5a**–**r**.

The target compounds **5a**–**r** (Scheme 1) were synthesized using previously reported literature methods [38,39]. The required aromatic azides **2a**–**e** and benzyl or alkyl azides **2f**–**r** were first synthesized by the reaction of aromatic anilines **1a**–**e** with dil. HCl and NaNO_2_ in water at 0–5 °C, followed by the addition of sodium azide in water at room temperature. The benzyl and other alkyl bromides **1f**–**r** were made to react with sodium azide to give azide reactants **2f**–**r** by the replacement of the terminal bromide. Finally, Cu(I) catalyzed coupling reactions between 6-chloro-3-methyl-1-(prop-2-yn-1-yl)pyrimidine-2,4(1*H*,3*H*)-dione (**4**) and the aromatic/benzyl/alkyl azides **2a**–**r** was achieved by refluxing in DMF at 80 °C for 12 h to produce the target compounds **5a**–**r**. As a representative case, the spectral analysis of compound **5e** is discussed below.

The formation of the substituted triazole-uracil compound **5e** was confirmed by analyzing its 400 MHz ^1^H-NMR spectrum (Supplementary Materials, Figure S10) which was recorded in CDCl_3_. The protons of the –CH_2_– group bridging between the uracil and triazole rings appeared at δ (ppm) 5.44 (s, 2H). The uracil ring proton was present at δ 6.02 (s, 1H) and phenyl ring protons appeared at δ 7.65 (m, 2H) and 7.54 (m, 2H). The methoxy protons of the phenyl ring and N–CH_3_ protons of uracil appeared at 3.94 (s, 3H) and 3.34 (s, 3H), respectively. The triazole ring proton appeared at δ 8.08 (s, 1H). The 100.6 MHz ^13^C-NMR spectrum of the compound was also recorded in CDCl_3_, (Supplementary Materials, Figure S11). The uracil carbonyl carbons appeared at δ 164.3 (C_2_) and δ 150.5 (C_5_). The triazole ring carbons appeared at δ 134.0 and 126.9. The four phenyl carbons were assigned to the peaks at δ 160.5 (C4′), 131.9 (C1′), 131.3 (C2′), 114.7 (C3′). The carbon of the –CH_2_– group between the uracil and triazole rings was observed at δ 36.1. The methoxy and N–CH_3_ carbons appeared at δ 66.6 and δ 28.4, respectively. The peaks that appeared at *m*/*z* 348 and 349 in the mass spectrum of **5e** (Figure S37) were assigned to the [M + H]^+^ and [M + 2]^+^ molecular ion peaks, respectively.

### 2.2. In Silico Docking Study

**Figure 2:**
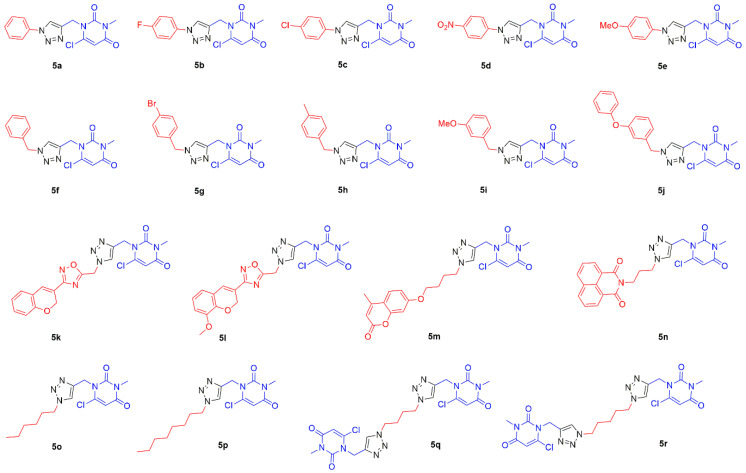
Picture showing all the substituted 1,2,3-triazole-uracil ensembles that were docked into the active site of the VEGFR-2 receptor protein.

**Figure 3:**
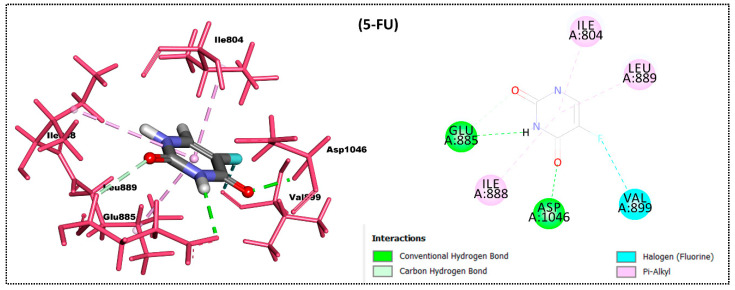
Docking interactions (3D & 2D) of reference drug **5-FU** with the receptor protein, VEGFR-2 transferase. Critical amino acid residues are shown in the stick model and pink colour.

**Figure 4:**
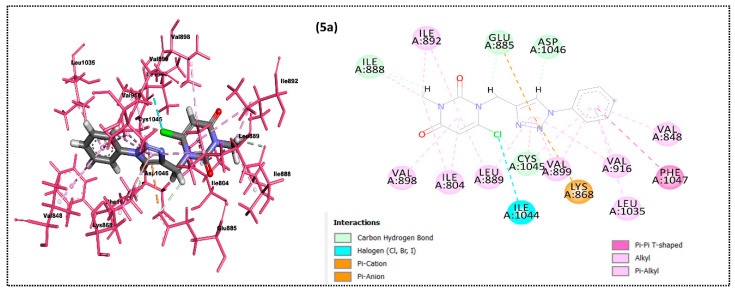
Docking interactions (3D & 2D) of **5a** with the receptor protein, VEGFR-2 transferase. Critical amino acid residues are shown in the stick model and pink colour.

**Figure 5:**
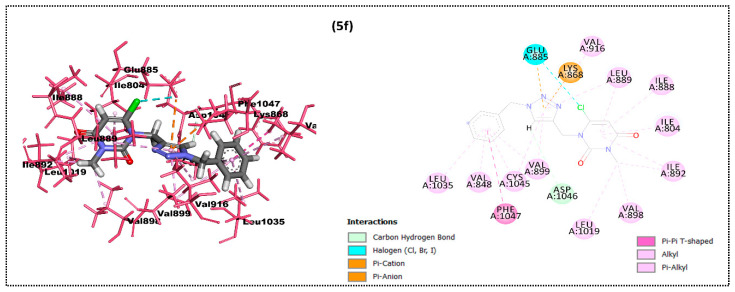
Docking interactions (3D & 2D) of **5f** with the receptor protein, VEGFR-2 transferase. Critical amino acid residues are shown in the stick model and pink colour.

**Figure 6:**
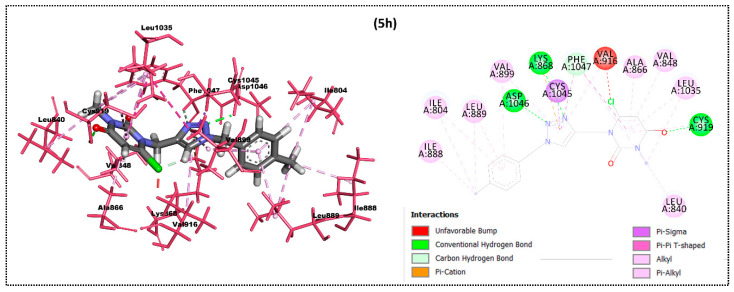
Docking interactions (3D & 2D) of **5h** with the receptor protein, VEGFR-2 transferase. Critical amino acid residues are shown in the stick model and pink colour.

**Figure 7:**
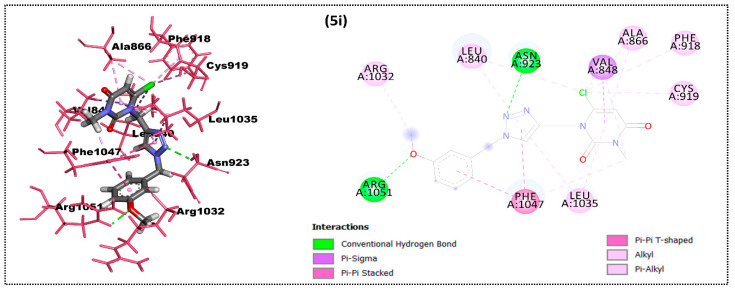
Docking interactions (3D & 2D) of **5i** with the receptor protein, VEGFR-2 transferase. Critical amino acid residues are shown in the stick model and pink colour.

**Figure 8:**
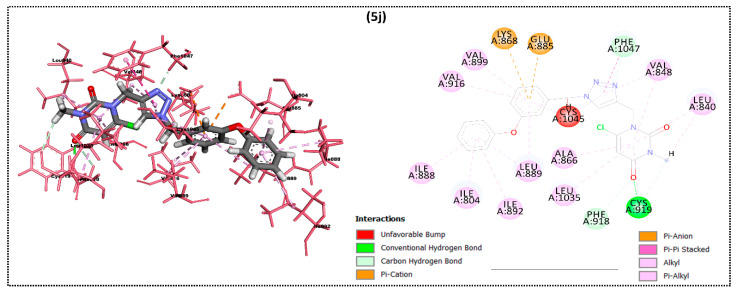
Docking interactions (3D & 2D) of **5j** with the receptor protein, VEGFR-2 transferase. Critical amino acid residues are shown in the stick model and pink colour.

**Figure 9:**
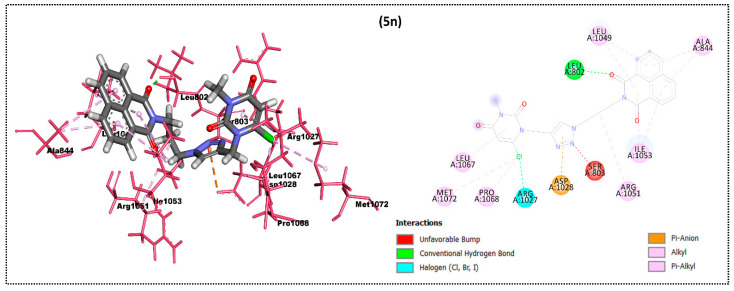
Docking interactions (3D & 2D) of **5n** with the receptor protein, VEGFR-2 transferase. Critical amino acid residues are shown in the stick model and pink colour.

**Figure 10:**
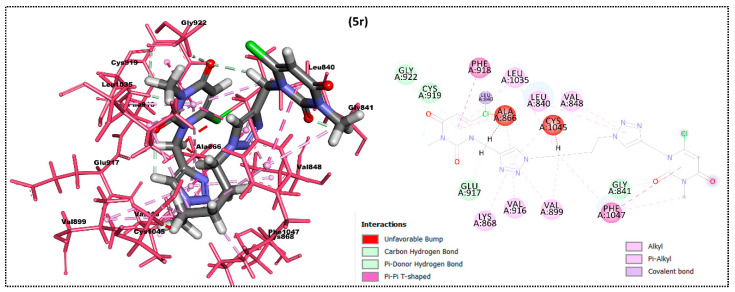
Docking interactions (3D & 2D) of 5r with the receptor protein, VEGFR-2 Transferase. Critical amino acid residues are shown in the stick model and pink colour.

Molecular docking is an essential tool to screen the binding affinities and mode of binding of protein-ligand interactions [40]. The docking analysis of the synthesized ligands (Figure 2) **5a**–**r** was performed using the Molegro virtual docker. A well-established molecular target, VEGFR-2 (PDB ID: 4AGD), was selected to study the docking interactions. The active site pocket of the receptor protein consists of Leu840, Gly922, Leu1038, Phe1047, Asp1046, Glu885, Val916, Glu917, Phe918, Cys919, Val848, Val899, Ile392, Leu889, Val916, Lys868, Gly841, Cys1045, Asp1052, Lys1055, Tyr1082, Arg1027, Leu802, Ser803, Leu1053, Pro1068, Tyr1054 and Arg842 amino acid residues. The results (Table 1) indicated that all the compounds bound to VEGFR-2 through hydrogen bonds and Van der Waal interactions with high binding affinities ranging from −108 to −198.7 kcal/mol. Figure 3, Figure 4, Figure 5, Figure 6, Figure 7, Figure 8, Figure 9 and Figure 10 depict the best binding poses for the reference drug **5-FU** and compounds **5a**, **5f**, **5h**, **5i**, **5j**, **5n** and **5r**.

The reference drug, **5-FU** produced a binding affinity of −65.73 kcal/mol. The interactions of the reference drug with the target protein are depicted in Figure 3. The –NH and carbonyl groups of the uracil moiety were stabilized by conventional hydrogen bonds with amino acid residues Glu885 (2.37 Å) and Asp1046 (2.03 Å), respectively. The fluorine atom of the uracil core interacted with the amino acid Val899. Further, the uracil nucleus is anchored by π-alkyl interactions with the amino acids Ile888, Ile804 and Leu889.

The synthesized compound **5a** showed a binding affinity of −108.00 kcal/mol and the corresponding interactions are presented in Figure 4. The uracil core is anchored by π-alkyl interactions with the Ile804, Val898, Ile892 and Leu889 residues. The chlorine atom of the uracil ring interacted with the carbonyl backbone of the Ile1044 amino acid. The methyl group present on the nitrogen of the uracil ring interacted with the carbonyl oxygen of Ile888. However, the carbonyl oxygens of the uracil ring did not form any kind of interactions with the target protein. The triazole ring is located in the hydrophobic pocket formed by Val899, Cys1045, Lys868 and Leu889 with π-alkyl interactions. The hydrogen atom of the triazole ring is bonded to the carbonyl oxygen of the Asp1046 residue. Further, the triazole moiety is stabilized by π-cation and π-anion interactions with the amino acids Lys868 and Glu885, respectively. The appended phenyl ring of **5a** showed π-π interactions with Phe1047 and π-alkyl interactions with the Val848, Val916, Val899, Leu1035 and Cys1045 amino acid residues.

Compound **5f** produced a binding affinity of −112.05 kcal/mol and the corresponding interactions are presented in Figure 5. The uracil moiety is anchored by π-alkyl interactions with the Leu889, Ile804, Ile888 and Ile892 residues. The chlorine atom of the uracil ring interacted with the carboxyl group of the Glu885 amino acid. The methyl group present on the nitrogen of the uracil ring settled in the hydrophobic pocket formed by the Ile892, Val898 and Leu1019 residues. The triazole ring is located in the hydrophobic pocket formed by Val916, Leu889, Val899 and Cys1045 with π-alkyl interactions. The hydrogen atom of the triazole ring is bonded to the carbonyl oxygen of the Asp1046 residue. Further, the triazole moiety is stabilized by π-cation and π-anion interactions with the amino acids Lys868 and Glu885, respectively. The appended benzyl ring of **5f** is stabilized via π-π interactions with Phe1047 and π-alkyl interactions the with amino acid residues Val848, Val899, Leu1035 and Cys1045.

Compound **5h** showed a binding affinity of −124.79 kcal/mol and the corresponding interactions are presented in Figure 6. It produced three conventional hydrogen bonds in the active region of the target protein. The carbonyl oxygen of the uracil ring formed a hydrogen bond with Cys919 with a bond distance of 1.97 Å. The two nitrogens at the 2 and 3-positons of the triazole ring formed hydrogen bonds with Asp1046 (2.06 Å) and Lys868 (2.54 Å), respectively. The uracil core is anchored by π-alkyl interactions with the Ala868, Val848, Cys919 and Leu1035 residues. The methyl group present on the nitrogen of the uracil ring interacted with the alkyl groups of the amino acids Leu840 and Leu1035 and it also formed a π-sigma interaction with the Phe1047 residue. The triazole ring is anchored by π-alkyl interactions with Val916, Cys1045 and Val899. Further, the triazole core is stabilized byπ-π and π-cation interactions with the amino acids Phe1047 and Lys868, respectively. The appended benzyl ring of **5h** showed π-alkyl interactions with the amino acids Ile804, Leu889 and Val899. The methyl group present on this phenyl ring is located in the hydrophobic pocket formed by the Ile888, Ile804 and Leu889 residues.

Compound **5i** showed a binding affinity of −118.52 kcal/mol and the corresponding interactions are depicted in Figure 7. It showed three conventional hydrogen bonds in the active site of the receptor protein. The nitrogen at the 2-positon of the triazole ring formed a hydrogen bond with Asn923 with a bond distance of 2.48 Å. The oxygen of the appended benzyl group produced two hydrogen bonds with Arg1051 forming a stable six-membered ring with bond distances of 2.16 and 2.37 Å. The chlorine atom of the uracil moiety produced four hydrophobic interactions with the amino acids Leu840, Cys919, Phe918 and Ala866. The uracil core is anchored by π-alkyl interactions with Leu1035, Ala866 and Val848. N-methyl group of uracil ring interacted with Val848 and Phe1047 via alkyl and π-alkyl interactions. The triazole ring is stabilized by Leu1035 and Leu840 though π-alkyl interactions and Phe1047 with π-π interactions. The appended benzyl ring showed a π-π interaction with the amino acid Phe1047.

Compound **5j** produced a binding affinity of −152.32 kcal/mol and the corresponding interactions are presented in Figure 8. It produced two hydrogen bonds in the active region of the target protein. The carbonyl oxygen of the uracil ring formed two hydrogen bonds with Cys919 and Phe918 with bond distances of 1.61 and 2.93 Å, respectively. The uracil core is anchored by π-alkyl interactions with Ala866, Val848, leu1035, Cys919 and Lue840. The N-methyl group of the uracil ring interacted with the Leu840 and Cys919 residues via alkyl interactions. The triazole ring is stabilized by π-alkyl interactions with Cys1045 and Val848 and π-π interactions with Phe1047. The appended benzyl ring showed a π-alkyl interaction with the amino acids Val916, Val899 and Leu889. Further, the benzyl ring is stabilized by π-cation and π-anion interactions with the amino acids Lys868 and Glu885, respectively. The phenoxy ring produced π-alkyl interactions with the amino acids Ile804, Leu889, Ile888 and Ile892.

Compound **5n** produced a binding affinity of −125 kcal/mol and the corresponding interactions are presented in Figure 9. It produced one conventional hydrogen bond with the target protein. The carbonyl oxygen of the isoquinoline ring formed one hydrogen bond with Leu802 with a bond distance of 1.96 Å. The uracil core is stabilized by a π-alkyl interaction with the amino acid Leu1067. The uracil chlorine atom produced two hydrophobic interactions with the Met1072 and Pro1068 amino acids and it also interacted with the carbonyl backbone of Arg1027. The triazole ring is stabilized by π-anionic interactions with the carboxylic acid group of Asp1028. The isoquinoline ring is anchored by π-alkyl interactions with the amino acids Ile1053, Leu1049 and Ala844.

Of all the docked compounds, **5r** showed the highest binding affinity (−198 kcal/mol) with the receptor protein. The interactions of the molecule **5r** with the target protein are depicted in Figure 10. In this molecule, the uracil and triazole rings are anchored by π-π stacking interactions between uracil-triazole, triazole-triazole rings and uracil-Phe1047 amino acid residue of the receptor protein. The carbonyl oxygen of two uracil rings interacted with the side chain hydrogens of the amino acids Gly922 and Gly841. Further, the uracil and triazole rings are stabilized by the amino acids Leu1035, Leu840, Val848, Lys868, Val916 and Val899 through π-alkyl interactions.

### 2.3. ADME Properties

A favorable absorption, distribution, metabolism and elimination profile is a prerequisite for any drug candidate to conclude its drug-likeness. The ADME properties for the reported ligands, were predicted using the Lipinski filters from the Molinspiration online tool kit. The properties like molecular weight, molecular volume, the logarithm of partition coefficient, hydrogen bond acceptors and donors, polar surface area, the number of rotatable bonds and Lipinski’s rule of five were determined by using the Molinspiration online property calculation toolkit [41]. Absorption percentage was obtained by: %ABS = 109 − (0.345 × TPSA) [42].

The Molsoft software was utilized to calculate properties like physico-chemical parameters, pharmacokinetics, pharmacodynamics (drug-likeness model score) and was represented by a numerical value [43]. The pharmacokinetic parameters for the synthesized ligands are presented in Table 2.

The results indicated good %Abs values for the triazole ligands ranging from 57.43% to 83.21%. Furthermore, compounds **5a**–**r** obeyed Lipinski’s rule of five (number of hydrogen bond acceptors (*n*-ON) ≤ 10) and followed the criteria for orally active drug and therefore, these compounds may have a good potential for eventual development as oral agents. A molecule likely to be developed as an orally active drug candidate should not produce more than one violation of the following four criteria: m_i_LogP (octanol-water partition coefficient) ≤ 5, molecular weight ≤ 500, number of hydrogen bond acceptors ≤ 10 and number of hydrogen bond donors ≤ 5 [44]. All the synthesized triazole-uracil analogues **5a**–**r** obeyed the criteria and hence, can be considered for the development as orally active drugs.

### 2.4. In Vitro Cytotoxicity

**Figure 11:**
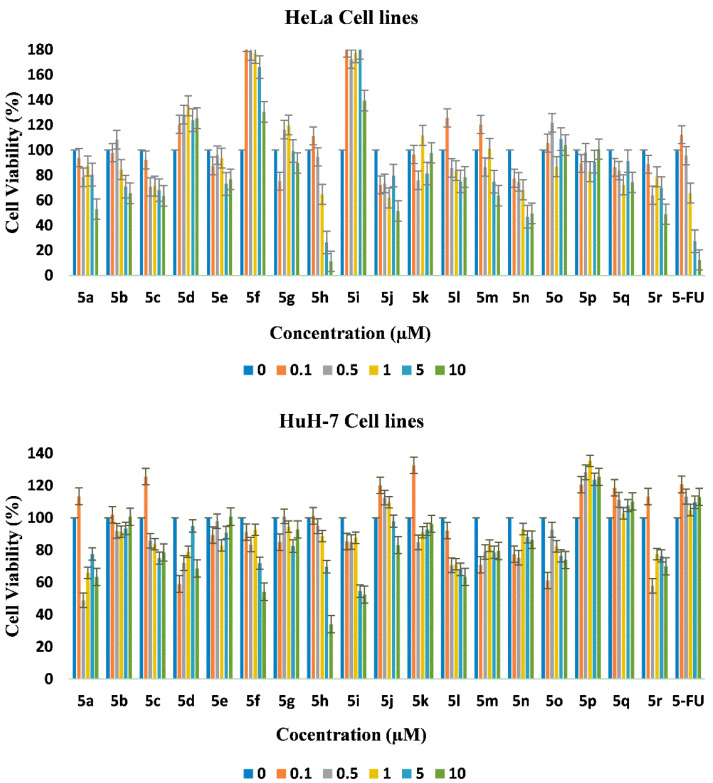
Cell viability profiles of HeLa and HUH-7 tumor cell lines against the treatment of substituted 1,2,3-triazole-uracil analogues for 72 h.

The cytotoxic potency for **5a**–**r** was assayed against HeLa and HUH-7 tumor cells by the standard MTT protocol. The ligands significantly inhibited the proliferation of the selected tumor cells and the corresponding viability profiles are presented in Figure 11.

The half-maximal inhibitory concentrations (IC_50_) for compounds **5a**–**r** are presented in Table 3. The ligands **5a**, **5h**, **5i**, **5j**, **5n** and **5r** produced higher inhibition effect against HeLa cells, with IC_50_ values ranging from 4.5 μM to 11.2 μM. HUH-7 cells were found to be more sensitive towards the treatment of **5f**, **5h** and **5i** with IC_50_ values 10.8 μM, 7.7 μM and 9 μM, respectively, and the remaining compounds failed to hamper the proliferation of HUH-7 tumor cells. Compound **5h** emerged as a hit compound and produced IC_50_ values of 4.5 μM and 7.7 μM, respectively, against both the cell lines. The obtained values for **5h** are better than those of the standard drug, 5-FU (4.6 μM and 30 μM) and it has the potential to be developed as a new chemotherapeutic agent for cervical and liver carcinoma.

For all the compounds **5a**–**r**, the 1,2,3-triazole and uracil rings are common, and hence some structure-activity relationship inferences can be drawn based on the different substituents. The compounds **5b**–**e** bearing a phenyl ring with electron-donating or withdrawing groups at the *para* position showed lower activity than the unsubstituted compound **5a**. The compounds **5f**–**j** (except **5g**) bearing a benzyl ring and with various electron-donating groups produced higher activity against HeLa or HUH-7 cells. The compounds **5k**–**m** with chromene rings failed to show any remarkable activity against the selected tumor cell lines. The compound **5n** with an isoquinoline ring showed good activity against HeLa cells. Compounds **5o** and **5p** with aliphatic hydrocarbon chains did not induce considerable activity against either of the cancer cell lines. The compound **5r** produced a higher inhibition effect than its dimer analogue **5q**. Interestingly, the compounds **5a**–**r** did not show any cytotoxicity towards the normal NIH/3T3 cells and this offer a great future for the reported ligands in cancer research.

On the other hand, the approved drugs for the treatment of liver cancer are sparse and sorafenib (VEGFR inhibitor) is the only drug approved to treat patients with advanced hepatocellular carcinoma [45,46,47]. Even though it is effective in treating liver cancer, patients are reported to develop resistance within six months and no alternative treatment is currently available [48,49]. Thus, the current results, that are close to the in vitro cytotoxicity of sorafenib [22,28] against liver carcinoma, are exciting and the reported ligands **5f**, **5h** and **5i** could be developed as new alternatives for treating liver cancer.

## 3. Materials and Methods

### 3.1. General Information

All the chemicals used in this study were obtained from Sigma Chemicals (St. Louis, MO, USA). Solvents of reagent grade were purchased from nearby commercial sources and distilled before the usage. DMEM media and the serum supplement FBS for the cytotoxicity assay were obtained from Sigma Chemicals and Gibco (Hyderabad, TS, India), respectively. The melting points of the ligands were measured in open capillaries using the electrothemal melting point apparatus. ^1^H- and ^13^C-NMR spectra were recorded using Avance II 400 and 100 MHz spectrometers (Bruker, Zurich, Switzerland) in 99.99% DMSO-*d*_6_ and 99.82% CDCl_3_ (2 mg/mL) using TMS as the standard solvent. ESI-MS spectra were measured on a LCMS 2010 VG mass spectrometer (Thermo Finnigan, San Jose, CA, USA) in methanol/DCM solvents (10 μg/mL). Elemental analysis was carried out using a Carlo Erba 1108 analyzer (Heraeus, Hanau, Germany).

### 3.2. Syntheses

#### 3.2.1. Synthesis of 6-Chloro-3-methyl-1-(prop-2-yn-1-yl)pyrimidine-2,4(1*H*,3*H*)-dione (**4**)

A DMF (50 mL) solution of **3** (1 g, 0.0062 mol) was slowly added with propargyl bromide (1.33 mL, 0.015 mol) and anhydrous K_2_CO_3_ (2.56 g, 0.0186 mol). The resulting mixture was allowed to reflux for **4** h and the course of the reaction was monitored by TLC until its completion. When the reaction was complete, the remaining solvent was evaporated under vacuum. Excess propargyl bromide was removed by washing the reaction mixture with hexane and then poured into ice-water (100 mL) and extracted with ethyl acetate. Then, the organic layer was added with the brine solution and residual water content after workup was removed by sodium sulphate. Final evaporation under high vacuum and purification by column chromatography produced the desired compound **4**.

White solid; 92% yield; MP 120.3–121.5 °C; ^1^H-NMR (400 MHz, CDCl_3_), δ, ppm: 5.99 (s, 1H), 4.86 (d, 2H, *J* = 2.0 Hz), 3.35 (s, 3H), 2.39 (s, 1H). M.F. C_8_H_7_ClN_2_O_2_; M. Wt. Cal: 198. Elemental analysis: Cal: C, 48.38; H, 3.55; N, 14.11; Found: C, 48.35; H, 3.50; N, 14.05.

#### 3.2.2. Synthesis of Substituted 1,2,3-Triazole-uracil Scaffolds **5a**–**r**

To a mixture of 6-chloro-3-methyl-1-(prop-2-yn-1-yl)pyrimidine-2,4(1*H*,3*H*)-dione (**4**) and alkyl/benzyl/aryl azides **2a**–**r** in DMF and water (1:1) solution, copper sulphate pentahydrate (2 mol%) and sodium ascorbate (10 mol%) were added. The reaction mixture was stirred for 1–1.5 h to afford **5a**–**r**.

*6-Chloro-3-methyl-1-((1-phenyl-1H-1,2,3-triazol-4-yl)methyl)pyrimidine-2,4(1H,3H)-dione* (**5a**): Yellow solid; 87% yield; M.P. 120–121 °C; ^1^H-NMR (400 MHz, CDCl_3_), δ, ppm: 8.11 (s, 1H), 7.74–7.72 (d, 2H, *J* = 7.2 Hz), 7.55–7.49 (m, 3H), 5.97 (s, 1H), 5.44 (s, 2H), 3.34 (s, 3H). ^13^C-NMR (100.6 MHz, CDCl_3_), δ, ppm: 160.6, 151.1, 145.3, 136.8, 129.8, 129.0, 120.6, 102.4, 41.9, 28.3. M.F. C_14_H_12_ClN_5_O_2_; M. Wt. Cal: 317; Found: ESI-Ms *m*/*z*, 318 [M + H]^+^. Elemental analysis: Cal: C, 52.92; H, 3.81; N, 22.04; Found: C, 52.86; H, 3.93; N, 22.15.

*6-Chloro-1-((1-(4-fluorophenyl)-1H-1,2,3-triazol-4-yl)methyl)-3-methylpyrimidine-2,4(1H,3H)-dione* (**5b**): Light white solid; 87% yield; M.P. 97 °C; ^1^H-NMR (400 MHz, CDCl_3_), δ, ppm: 8.08 (s, 1H), 7.69–7.66 (d, 2H, *J* = 8.8 Hz), 7.52–7.49 (d, 2H, *J* = 8.8 Hz), 5.98 (s, 1H), 5.43 (s, 2H), 3.34 (s, 3H). ^13^C-NMR (100.6 MHz, CDCl_3_), δ, ppm: 160.6, 151.1, 145.2, 142.9, 137.6, 135.6, 130.9, 129.1, 121.8, 120.8, 118.5, 102.5, 41.8, 28.3. M.F. C_14_H_11_ClFN_5_O_2_; M. Wt. Cal: 335; Found: ESI-Ms *m*/*z*, 358 [M + Na]^+^. Elemental analysis: Cal: C, 50.09; H, 3.30; N, 20.86; Found: C, 50.18; H, 3.23; N, 20.72.

*6-Chloro-1-((1-(4-chlorophenyl)-1H-1,2,3-triazol-4-yl)methyl)-3-methylpyrimidine-2,4(1H,3H)-dione* (**5c**): White solid; 85% yield; M.P. 100 °C; ^1^H-NMR (400 MHz, CDCl_3_), δ, ppm: 8.08 (s, 1H), 7.69–7.67 (d, 2H, *J* = 8.8 Hz), 7.51–7.49 (d, 2H, *J* = 8.8 Hz), 5.98 (s, 1H), 5.44 (s, 2H), 3.34 (s, 3H). ^13^C-NMR (100.6 MHz, CDCl_3_), δ, ppm: 160.6, 151.1, 145.2, 135.2, 134.8, 130.0, 121.3, 118.5, 102.5, 96.1, 41.8, 28.3. M.F. C_14_H_11_Cl_2_N_5_O_2_; M. Wt. Cal: 351; Found: ESI-Ms *m*/*z*, 352 [M + H]^+^. Elemental analysis: Cal: C, 47.75; H, 3.15; N, 19.89; Found: C, 47.89; H, 3.09; N, 19.82.

*6-Chloro-3-methyl-1-((1-(4-nitrophenyl)-1H-1,2,3-triazol-4-yl)methyl)pyrimidine-2,4(1H,3H)-dione* (**5d**): Brick red; 75% yield; M.P. 110–112 °C; ^1^H-NMR (400 MHz, CDCl_3_), δ, ppm: 8.43–8.41 (d, *J* = 9.1 Hz, 2H), 8.23 (s, 1H), 7.99–7.97 (d, *J* = 9.2 Hz, 2H), 6.00 (s, 1H), 5.46 (s, 2H), 3.35 (s, 3H). ^13^C-NMR (100.6 MHz, CDCl_3_), δ, ppm: 194.6, 160.5, 145.1, 143.5, 140.8, 126.5, 125.9, 121.8, 120.6, 102.6, 41.7, 28.3. M.F. C_14_H_11_ClN_6_O_4_; M. Wt. Cal: 362; Found: ESI-Ms *m*/*z*, 363 [M + H]^+^. Elemental analysis: Cal: C, 46.36; H, 3.06; N, 23.17; Found: C, 46.45; H, 3.14; N, 23.17.

*6-Chloro-1-((1-(4-methoxyphenyl)-1H-1,2,3-triazol-4-yl)methyl)-3-methylpyrimidine-2,4-(1H,3H)-dione* (**5e**): Yellow solid; 80% yield; M.P. 145–148 °C; ^1^H NMR (400 MHz, CDCl_3_), δ, ppm: 8.08 (s, 1H), 7.65 (d, 2H, *J* = 8.2 Hz), 7.54 (d, 2H, *J* = 8.4 Hz), 6.02 (s, 1H), 5.44 (s, 2H), 3.94 (s, 3H), 3.34 (s, 3H). ^13^C-NMR (100.6 MHz, CDCl_3_), δ, ppm: 164.3, 160.5, 150.5, 144.7, 134.0, 131.9, 131.3, 126.9, 114.7, 102.6, 66.6, 36.1, 28.4. M.F. C_15_H_14_ClN_5_O_3_; M. Wt. Cal: 347; Found: ESI-Ms *m*/*z*, 348 [M + H]^+^. Elemental analysis: Cal: C, 51.81; H, 4.06; N, 20.14; Found: C, 51.79; H, 4.01; N, 20.10.

*1-((1-Benzyl-1H-1,2,3-triazol-4-yl)methyl)-6-chloro-3-methylpyrimidine-2,4(1H,3H)-dione* (**5f**): Light yellow solid; 92% yield; M.P. 115–118 °C; ^1^H-NMR (400 MHz, CDCl_3_), δ, ppm: 8.59 (dd, *J* = 7.3, 0.9 Hz, 1H), 8.24 (dd, *J* = 8.3, 0.9 Hz, 2H), 7.81–7.72 (m, 2H), 6.91 (d, *J* = 8.7 Hz, 1H), 5.98 (s, 1H), 4.85 (d, *J* = 2.5 Hz, 2H), 4.43 (s, 2H), 3.35 (s, 3H). ^13^C-NMR (100.6 MHz, CDCl_3_), δ, ppm: 190.8, 164.3, 160.5, 144.7, 134.0, 131.9, 131.3, 126.9, 114.7, 102.6, 73.3, 36.1, 28.4. M.F. C_15_H_14_ClN_5_O_2_; M. Wt. Cal: 331; Found: ESI-Ms *m*/*z*, 332 [M + H]^+^. Elemental analysis: Cal: C, 54.30; H, 4.25; N, 21.11; Found: C, 54.36; H, 4.30; N, 21.18.

*1-((1-(4-Bromobenzyl)-1H-1,2,3-triazol-4-yl)methyl)-6-chloro-3-methylpyrimidine-2,4(1H,3H)-dione* (**5g**): Pale green; 81% yield; M.P. 138–140 °C; ^1^H-NMR (400 MHz, CDCl_3_), δ, ppm: 7.58 (s, 1H), 7.51 (dd, *J* = 8.5, 2.4 Hz, 2H), 7.18 (dd, *J* = 15.3, 8.4 Hz, 2H), 5.93 (s, 1H), 5.46 (s, 2H), 5.32 (s, 2H), 3.30 (s, 3H). ^13^C-NMR (100.6 MHz, CDCl_3_), δ, ppm: 160.6, 151.0, 145.3, 142.5, 134.4, 132.3, 131.9, 129.8, 123.3, 102.3, 53.5, 41.9, 28.3. M.F. C_15_H_13_ClBrN_5_O_2_; M. Wt. Cal: 409; Found: ESI-Ms *m*/*z*, 410 [M + H]^+^. Elemental analysis: Cal: C, 43.87; H, 3.19; N, 17.05; Found: C, 43.80; H, 3.15; N, 17.01.

*6-Chloro-3-methyl-1-((1-(4-methylbenzyl)-1H-1,2,3-triazol-4-yl)methyl)pyrimidine-2,4(1H,3H)-dione* (**5h**): Off white solid; 78% yield; M.P. 110–112 °C; ^1^H-NMR (400 MHz, CDCl_3_), δ, ppm: 7.53 (s, 1H), 7.18 (d, *J* = 7.2 Hz, 4H), 5.93 (s, 1H), 5.46 (s, 2H), 5.31 (s, 2H), 3.30 (s, 3H), 2.35 (s, 3H). ^13^C-NMR (100.6 MHz, CDCl_3_), δ, ppm: 160.7, 151.0, 145.4, 138.8, 131.2, 130.5, 129.8, 129.5, 128.5, 128.2, 102.3, 54.1, 41.9, 28.3, 21.1. M.F. C_16_H_16_ClN_5_O_2_; M. Wt. Cal: 345. Elemental analysis: Cal: C, 55.58; H, 4.66; N, 20.25; Found: C, 55.50; H, 4.60; N, 20.20.

*6-Chloro-1-((1-(3-methoxybenzyl)-1H-1,2,3-triazol-4-yl)methyl)-3-methylpyrimidine-2,4(1H,3H)-dione* (**5i**): Light yellow solid; 80% yield; M.P. 98–100 °C; ^1^H-NMR (400 MHz, CDCl_3_), δ, ppm: 7.57 (s, 1H), 7.29 (m, 2H, *J* = 7.2 Hz), 6.86 (m, 2H, *J* = 7.1 Hz), 5.93 (s, 1H), 5.46 (s, 2H), 5.32 (s, 2H), 3.81 (d, *J* = 2.4 Hz, 3H), 3.30 (s, 3H). ^13^C-NMR (100.6 MHz, CDCl_3_), δ, ppm: 190.8, 164.3, 160.5, 144.7, 134.0, 131.9, 131.3, 126.9, 114.7, 102.6, 73.3, 66.6, 37.7, 36.1, 35.2, 28.4. M.F. C_16_H_16_ClN_5_O_3_; M. Wt. Cal: 361. Elemental analysis: Cal: C, 53.12; H, 4.46; N, 19.36; Found: C, 53.08; H, 4.40; N, 19.30.

*6-Chloro-3-methyl-1-((1-(3-phenoxybenzyl)-1H-1,2,3-triazol-4-yl)methyl)pyrimidine-2,4(1H,3H)-dione* (**5j**): Cream yellow solid; 86% yield; M.P. 101–105 °C; ^1^H-NMR (400 MHz, CDCl_3_ + DMSO), δ, ppm: 8.57 (d, *J* = 7.3 Hz, 2H), 8.43 (d, *J* = 8.2 Hz, 2H), 8.07 (s, 1H), 7.86 (t, *J* = 7.8 Hz, 2H), 7.46 (d, *J* = 8.9 Hz, 2H), 7.06 (d, *J* = 8.9 Hz, 2H), 4.84 (s, 4H), 3.34 (s, 3H). M.F. C_21_H_18_ClN_5_O_3_; M. Wt. Cal: 423; Found: ESI-Ms *m*/*z*, 424 [M + H]^+^. Elemental analysis: Cal: C, 59.51; H, 4.28; N, 16.52; Found: C, 59.50; H, 4.21; N, 16.45.

*1-((1-((3-(2H-Chromen-3-yl)-1,2,4-oxadiazol-5-yl)methyl)-1H-1,2,3-triazol-4-yl)methyl)-6-chloro-3-methylpyrimidine-2,4(1H,3H)-dione* (**5k**): Off white solid; 86% yield; M.P. 125–128 °C; ^1^H-NMR (400 MHz, CDCl_3_), δ, ppm: 7.97 (s, 1H), 7.44 (s, 1H), 7.25 (d, 1H, J = 5.7 Hz), 7.16–6.93 (t, J = 7.5 Hz, 2H), 6.89 (d, 2H, J = 8.2 Hz), 5.97 (s, 1H), 5.84 (s, 2H), 5.40 (s, 2H), 5.16 (s, 2H), 3.32 (s, 3H). ^13^C-NMR (100.6 MHz, CDCl_3_), δ, ppm: 171.4, 166.2, 160.6, 154.6, 151.1, 145.2, 143.0, 131.4, 129.5, 128.4, 124.6, 121.9, 120.9, 118.1, 116.1, 102.4, 64.1, 45.1, 41.7, 28.3. M.F. C_20_H_16_ClN_7_O_4_; M. Wt. Cal: 453. Elemental analysis: Cal: C, 52.93; H, 3.55; N, 21.60; Found: C, 52.89; H, 3.50; N, 21.51.

*6-Chloro-1-((1-((3-(8-methoxy-2H-chromen-3-yl)-1,2,4-oxadiazol-5-yl)methyl)-1H-1,2,3-triazol-4-yl)methyl)-3-methylpyrimidine-2,4(1H,3H)-dione* (**5l**): Off white solid; 78% yield; M.P. 122–125 °C; ^1^H-NMR (400 MHz, CDCl_3_), δ, ppm: 7.96 (s, 1H), 7.44 (s, 1H), 6.90–6.75 (m, 3H), 5.97 (s, 1H), 5.84 (s, 2H), 5.39 (s, 2H), 5.22 (s, 2H), 3.90 (s, 3H), 3.32 (s, 3H). ^13^C-NMR (100.6 MHz, CDCl_3_), δ, ppm: 171.4, 166.1, 160.6, 151.1, 147.9, 146.3, 145.2, 143.5, 143.0, 129.5, 124.5, 121.6, 120.5, 118.2, 114.0, 102.4, 64.4, 56.1, 45.0, 41.7, 28.3. M.F. C_21_H_18_ClN_7_O_5_; M. Wt. Cal: 483; Found: ESI-Ms m/z, 484 [M + H]^+^. Elemental analysis: Cal: C, 52.13; H, 3.75; N, 20.26; Found: C, 52.07; H, 3.69; N, 20.19.

*6-Chloro-3-methyl-1-((1-(4-((4-methyl-2-oxo-2H-chromen-7-yl)oxy)butyl)-1H-1,2,3-triazol-4-yl)methyl)pyrimidine-2,4(1H,3H)-dione* (**5m**): Off white solid; 80% yield; M.P. 145–148 °C; ^1^H-NMR (400 MHz, CDCl_3_), δ, ppm: 8.60 (dd, J = 7.3, 1.1 Hz, 2H), 8.25 (dd, J = 8.3, 1.0 Hz, 2H), 7.85 (s, 1H), 7.78 (dd, J = 8.2, 7.4 Hz, 2H), 5.94 (s, 1H), 5.35 (s, 2H), 4.48 (t, J = 7.2 Hz, 2H), 4.26 (t, J = 6.8 Hz, 3H), 3.33 (s, 3H), 2.46–2.35 (m, 2H), 2.05 (t, J = 3.5 Hz, 2H). ^13^C-NMR (100.6 MHz, CDCl_3_), δ, ppm: 164.3, 160.7, 151.1, 145.4, 141.9, 134.3, 134.1, 131.5, 127.0, 123.6, 122.3, 102.3, 48.3, 41.9, 37.4, 29.7, 29.1, 28.9, 28.3. M.F. C_22_H_22_ClN_5_O_5_; M. Wt. Cal: 471. Elemental analysis: Cal: C, 55.99; H, 4.70; N, 14.84; Found: C, 55.87; H, 4.64; N, 14.75.

*2-(3-(4-((6-Chloro-3-methyl-2,4-dioxo-3,4-dihydropyrimidin-1(2H)-yl)methyl)-1H-1,2,3-triazol-1-yl)propyl)-1H-benzo[de]isoquinoline-1,3(2H)-dione* (**5n**): White solid; 67% yield; M.P. 96–98 °C; ^1^H-NMR (400 MHz, CDCl_3_), δ, ppm: 8.60 (dd, J = 7.3, 0.9 Hz, 2H), 8.23 (dd, J = 8.3, 0.8 Hz, 2H), 7.82–7.72 (m, 2H), 7.67 (s, 1H), 5.96 (d, J = 15.1 Hz, 1H), 5.34 (s, 2H), 4.85 (d, J = 2.5 Hz, 1H), 4.37 (t, J = 7.3 Hz, 2H), 4.25–4.12 (m, 2H), 3.33 (s, 3H). ^13^C-NMR (100.6MHz, CDCl_3_), δ, ppm: 164.2, 160.7, 151.1, 145.4, 134.0, 131.6, 131.3, 128.1, 126.9, 123.3, 122.5, 102.6, 102.3, 73.3, 50.2, 42.0, 39.8, 36.1, 29.8, 28.3, 27.3, 23.9. M.F. C_23_H_19_ClN_6_O_4_; M. Wt. Cal: 478; Found: ESI-Ms m/z, 479 [M + H]^+^. Elemental analysis: Cal: C, 57.68; H, 4.00; N, 17.55; Found: C, 57.60; H, 4.09; N, 17.48.

*6-Chloro-1-((1-hexyl-1H-1,2,3-triazol-4-yl)methyl)-3-methylpyrimidine-2,4(1H,3H)-dione* (**5o**): Brown solid; 80% yield; M.P 100–102 °C; ^1^H-NMR (400 MHz, CDCl_3_), δ, ppm: 7.64 (s, 1H), 5.95 (s, 1H), 5.35 (s, 2H), 4.37–4.29 (m, 2H), 3.33 (s, 3H), 2.10–1.68 (m, 2H), 1.31 (d, *J* = 1.9 Hz, 6H), 0.88 (s, 3H). ^13^C-NMR (100.6 MHz, CDCl_3_), δ, ppm: 160.7, 152.5, 151.1, 145.4, 123.3, 102.3, 50.5, 42.0, 31.0, 30.1, 28.3, 26.1, 22.3, 13.9. M.F. C_14_H_20_ClN_5_O_2_; M. Wt. Cal: 325. Elemental analysis: Cal: C, 51.61; H, 6.19; N, 21.50; Found: C, 51.57; H, 6.12; N, 21.46.

*6-Chloro-3-methyl-1-((1-octyl-1H-1,2,3-triazol-4-yl)methyl)pyrimidine-2,4(1H,3H)-dione* (**5p**): Brown solid; 68% yield; M.P. 120–121 °C; ^1^H-NMR (400 MHz, CDCl_3_), δ, ppm: 7.62 (s, 1H), 5.95 (s, 1H), 5.35 (s, 2H), 4.32 (t, *J* = 7.3 Hz, 2H), 3.33 (s, 3H), 3.25 (t, *J* = 7.0 Hz, 3H), 1.63 (d, *J* = 4.0 Hz, 6H), 1.44 (d, *J* = 6.3 Hz, 2H), 0.88 (d, *J* = 7.1 Hz, 4H). ^13^C-NMR (100.6 MHz, CDCl_3_), δ, ppm: 160.7, 151.1, 145.4, 141.8, 123.2, 102.3, 51.5, 50.5, 42.0, 31.7, 30.2, 28.3, 26.7, 26.4, 22.6, 14.0. M.F. C_16_H_24_ClN_5_O_2_; M. Wt. Cal: 353. Elemental analysis: Cal: C, 54.31; H, 6.84; N, 19.79; Found: C, 54.36; H, 6.94; N, 19.71.

*1,1’-((Butane-1,4-diylbis(1H-1,2,3-triazole-1,4-diyl))bis(methylene))bis(6-chloro-3-methylpyrimidine-2,4(1H,3H)-dione)* (**5q**): Pale yellow solid; 83% yield; M.P. 115–118 °C; ^1^H-NMR (400 MHz, CDCl_3_), δ, ppm: 7.66 (s, 2H), 5.96 (s, 2H), 5.35 (s, 4H), 4.39 (t, *J* = 7.0 Hz, 4H), 3.42 (t, *J* = 6.4 Hz, 4H), 3.33 (s, 6H). ^13^C-NMR (100.6 MHz, CDCl_3_), δ, ppm: 160.6, 151.1, 145.4, 142.0, 123.3, 102.3, 51.0, 50.2, 41.9, 29.7, 28.2, 23.7. M.F. C_20_H_22_Cl_2_N_10_O_4_; M. Wt. Cal: 536; Found: ESI-Ms *m*/*z*, 537 [M + H]^+^. Elemental analysis: Cal: C, 44.70; H, 4.13; N, 26.07. Found: C, 44.65; H, 4.08; N, 26.01.

*1,1’-((Pentane-1,5-diylbis(1H-1,2,3-triazole-1,4-diyl))bis(methylene))bis(6-chloro-3-methylpyrimidine-2,4(1H,3H)-dione)* (**5r**): White solid; 90% yield; M.P. 114–115 °C; ^1^H-NMR (400 MHz, CDCl_3_), δ, ppm: 7.64 (s, 2H), 5.95 (s, 2H), 5.35 (s, 4H), 4.35 (t, *J* = 7.2 Hz, 4H), 3.33 (s, 6H), 3.28 (t, *J* = 6.7 Hz, 4H), 1.98–1.93 (m, 2H). ^13^C-NMR (100.6 MHz, CDCl_3_), δ, ppm: 160.6, 151.1, 145.4, 142.0, 123.3, 102.3, 63.3, 51.0, 50.2, 41.9, 29.7, 28.2, 23.7. M.F. C_21_H_24_Cl_2_N_10_O_4_; M. Wt. Cal: 551. Elemental analysis: Cal: C, 45.74; H, 4.39; N, 25.40; Found: C, 45.69; H, 4.32; N, 25.34

### 3.3. In Silico Docking Simulations

#### 3.3.1. Preparation of Ligands and Target Protein

The structures of all the ligands **5a**–**r** were drawn using the ChemDraw suite (Ver.14.0.0.117, PerkinElmer Inc., Waltham, MA, USA) [50] and energy minimization was accomplished by OPLS 2005 through the Ligprep module of the Schrodinger suite (Schrödinger, New York, NY, USA) [51]. Ionization states for ligands were retained at their original values and saved in .sdf format for docking simulations. The crystal structure of the VEGFR-2 protein was retrieved from the protein data bank (PDB ID: 4AGD). Molecular docking operations in the active site of receptor protein were executed by the Molegro virtual docking program (MVD 6.0, 2013, Molexus, Odder, Denmark). All the solvent molecules attached to the receptor protein were removed and structural parameters like hybridization state, bond order, explicit hydrogen atoms and relevant charges for ligands were assigned according to the docking software instructions. Five potential cavities for binding were obtained by using the detect cavities option from the preparation tools. The cavity, surrounding the anion binding site with a volume of 177 Å^3^, was further modified by side-chain minimization and used for virtual screening. Moldock score (grid-based) with a grid resolution of 0.20 Å was used as a scoring function for docking analysis [52]. Moldock and Rerank scores estimated the best binding poses for ligands. ALIGN program from COOT graphical program was used for molecular alignments [53].

#### 3.3.2. Molecular Docking

MVD is a convenient and fast algorithm to estimate protein and ligand interactions. The virtual screening of synthesized ligands depends on Rerank score which is a mathematical expression for protein-ligand binding affinity and is based on the Moldock score derived from PLP (piecewise linear potential) [14]. Total energy for the protein-ligand system was minimized by Medler Mead simplex minimization with H bond directionality and non-grid force field [54]. Binding affinities of ligands with protein were computed based on internal electrostatic, hydrogen bond interactions and sp^2^-sp^2^ tortions. All the docking interactions of the protein-ligand system were analyzed using Accelrys Discovery Studio v3.5 (Waltham, MA, USA) [55].

### 3.4. ADME Profiles

Drug-likeness of the derived ligands was assessed by Lipinski filters in the Molinspiration webserver (^©^Molinspiration Cheminformatics 2018, Nova Ulica, Slovensky Grob, Slovak Republic).

### 3.5. Softwares, Suites and Webservers

The ligands were designed by Marvin Sketch 5.6.0.2 (1998–2011; Copyright Chem Axon Ltd., San Diego, CA, USA). Docking interactions were obtained from Molegro Virtual Docker (2010.4.0.0). Accelrys Discovery studio visualizer 3.5.0.12158 (©2005–12; Accelrys Software Inc., San Diego, CA, USA) was utilized for molecular visualizations. Solubility parameters were calculated by the Qikprop module from Schrodinger Suite 2013.

### 3.6. In Vitro Cytotoxicity

The cell viability was measured by MTT colourimetric assay. The cell lines viz., HeLa (human cervical adenocarcinoma), HUH-7 (human hepatoma cancer cells) and NIH/3T3 (normal murine fibroblast cells) were supplemented with 10% fetal bovine serum and maintained in an environment of 5% CO_2_ at 37 °C temperature. Cell lines at a density of 2 × 10^3^ cells/well were seeded onto 96 well plates for 16 h and maintained in DMEM culture media. Five different concentrations of synthesized ligands and 5-fluorouracil (except for normal cells) i.e., 0.1, 0.5, 1, 5 and 10 μM were treated against each cell line and incubated for 3 days at 37 °C [56]. Then, the solutions were discarded and an MTT reagent with a final concentration of 500 μg/mL was added to each well and plate and incubated for another 4–6 h [57,58,59]. 200 μL of DMSO was added to solubilize purple precipitates (formazan product) and absorbance (optical density) was recorded at 570 nm on ELISA reader. IC_50_ values were calculated using dose-response data by applying the linear regression method. The experiments were done in triplicate for each concentration of ligand.

## 4. Conclusions

Herein, eighteen new 1,2,3-triazole-uracil scaffolds have been designed, synthesized and tested as anti-cancer agents. The structures of the compounds were established by standard analytical techniques viz., ^1^H-NMR, ^13^C-NMR, elemental analysis and mass spectrometry. The analytical data were in good agreement with the molecular formulae of the compounds. A rational approach of drug design was adopted to present the anti-tumor efficacies of the synthesized ligands **5a**–**r**. To realize the objective, the ligands **5a**–**r** were sequentially screened in the active site of VEGFR-2 protein and the results depicted remarkable interactions with the receptor protein. The molecular docking simulations by Molegro virtual docker produced high binding affinities for all the compounds (−108 to −198.7 kcal/mol) with various hydrophobic, electrostatic and hydrogen bond interactions. The interactions observed for the compounds **5a**, **5f**, **5h**, **5i**, **5j**, **5n** and **5r** determine proximity with receptor protein with different binding modes and justify their remarkable cytotoxic activity among all the synthesized compounds. The propinquity of ligands with receptor protein presumed the ability to inhibit in vitro cell proliferation and hence the ligands were screened for cell viability by the MTT method. Six compounds **5a**, **5h**, **5i**, **5j**, **5n** and **5r** were found to inhibit cell proliferation with low half-maximal inhibitory concentrations (IC_50_) of 11.2 μM, 4.5 μM**,** 9.0 μM, 10 μM, 9.6 μM, 9.6 μM, respectively. Compound **5h** emerged as a hit compound by producing higher inhibition activity than 5-FU against both the HeLa and HUH-7 tumor cell lines with IC_50_ values 4.5 and 7.7 μM, respectively. The docking results were very well correlated to the experimental results. Interestingly, the compounds didn’t show any cytotoxicity towards normal cells, paving the way for the reported ligands to be developed as new alternatives to existing drugs for cervical and liver cancers.

## Figures and Tables

**Table 1:** Moldock and rerank scores for compounds **5a**–**r**

Ligand	Moldock Score [Grid] (Kcal/mol)	Moldock Score	Rerank Score	RMSD
**5a**	−108.00	−111.498	−91.02	43.22
**5b**	−118.04	−121.41	−48.81	43.58
**5c**	−113.87	−117.85	−97.03	43.51
**5d**	−126.20	−129.04	−71.39	46.09
**5e**	−118.97	−118.2	−53.30	42.54
**5f**	−112.05	−113.68	−75.16	46.45
**5g**	−122.27	−117.88	−95.98	50.76
**5h**	−124.79	−126.60	−100.77	43.38
**5i**	−118.52	−119.90	−99.87	51.82
**5j**	−152.32	−155.11	−44.55	48.89
**5k**	−141.58	−147.40	−123.80	52.33
**5l**	−151.01	−155.75	−113.42	41.92
**5m**	−124.53	−124.14	−92.41	54.27
**5n**	−125.00	−112.56	−59.54	51.16
**5o**	−110.97	−111.43	−64.34	47.66
**5p**	−129.45	−126.51	−48.51	47.25
**5q**	−142.92	−128.58	−42.75	54.60
**5r**	−198.07	−204.56	−137.60	51.45
**5-FU**	−65.73	−65.92	−59.28	40.95

**Table 2:** Pharmacokinetic parameters of triazole-uracil scaffolds (**5a**–**r**).

C ^1^	Gpcr Ligand	Ion Channel Modulator	Kinase Inhibitor	Nuclear Receptor Ligand	Protease Inhibitor	Enzyme Inhibitor	milogP ^2^	TPSA (A2) ^3^	n Violation ^4^	M.wt ^5^	nO N ^6^	nOHNH ^7^	%ABS	MV ^8^
**Rule**							≤5	—	≤1	<500	<10	<5		
**5a**	0.11	−0.15	−0.21	−0.22	−0.28	0.09	1.28	74.73	0	317.74	7	0	83.21	259.51
**5b**	0.13	−0.16	−0.15	−0.17	−0.27	0.06	1.45	74.73	0	335.73	7	0	83.21	264.45
**5c**	0.12	−0.15	−0.20	−0.21	−0.27	0.06	1.96	74.73	0	352.18	7	0	83.21	273.05
**5d**	−0.04	−0.19	−0.32	−0.28	−0.35	−0.05	1.24	120.55	0	362.73	10	0	67.41	282.85
**5e**	0.07	−0.21	−0.21	−0.19	−0.27	0.03	1.34	83.96	0	347.76	8	0	80.03	285.06
**5f**	0.07	−0.19	−0.21	−0.26	−0.21	0.07	1.60	74.73	0	331.76	7	0	83.21	276.32
**5g**	−0.04	−0.26	−0.24	−0.36	−0.31	−0.01	2.41	74.73	0	410.66	7	0	83.21	294.20
**5h**	0.02	−0.25	−0.24	−0.28	−0.24	0.01	2.05	74.73	0	345.79	7	0	83.21	292.88
**5i**	0.07	−0.15	−0.11	−0.10	−0.24	0.01	1.64	83.96	0	361.79	8	0	80.03	301.86
**5j**	0.07	−0.15	−0.11	−0.10	−0.10	0.07	3.33	83.96	0	423.86	8	0	80.03	356.71
**5k**	0.06	−0.24	−0.34	−0.15	−0.19	0.01	1.45	122.89	0	453.85	11	0	66.60	363.34
**5l**	0.01	−0.28	−0.35	−0.22	−0.24	−0.03	1.26	132.12	1	483.87	12	0	63.41	388.89
**5m**	−0.20	−0.60	−0.36	−0.19	−0.34	0.02	2.66	114.17	0	471.90	10	0	69.63	396.81
**5n**	0.03	−0.29	−0.14	−0.32	−0.16	0.11	2.02	113.80	0	478.90	10	0	69.73	393.82
**5o**	0.12	−0.28	−0.29	−0.36	−0.18	0.16	2.46	74.73	0	325.80	7	0	83.21	288.68
**5p**	0.13	−0.26	−0.22	−0.26	−0.12	0.14	3.47	74.73	0	353.85	7	0	83.21	322.28
**5q**	0.07	−0.20	−0.12	−0.20	−0.09	0.08	0.24	149.46	2	551.39	14	0	57.43	430.79
**5r**	0.07	−0.23	−0.12	−0.19	−0.08	0.08	0.75	149.46	2	551.39	14	0	57.43	447.59
**5-FU**	−2.60	−1.95	−2.62	−3.04	−3.15	−1.56	−0.59	65.72	0	130.08	4	2	86.32	96.91

^1^ Compounds; ^2^ Measured lipophilicity; ^3^ Total polar surface area; ^4^ No of violations from Lipinski’s rule of five; ^5^ Molecular weight; ^6^ No. of hydrogen bond acceptors; ^7^ No. of hydrogen bond donors; ^8^ Molar volume.

**Table 3:** IC_50_ values (μM) for the new 1,2,3-triazole-uracil scaffolds **5a**–**r** against the treatment of HeLa and HUH-7 tumor cells and NIH/3T3 normal cells after 72 h.

Compound	HeLa	HUH-7	NIH/3T3
**5a**	11.2 ± 0.1	≫10	ND ^1^
**5b**	≫10	≫10	ND
**5c**	≫10	≫10	ND
**5d**	≫10	≫10	ND
**5e**	≫10	≫10	ND
**5f**	≫10	10.8 ± 0.5	ND
**5g**	≫10	≫10	ND
**5h**	4.5 ± 0.5	7.7 ± 0.3	ND
**5i**	≫10	9.0 ± 0.8	ND
**5j**	10 ± 1.5	≫10	ND
**5k**	≫10	≫10	ND
**5l**	≫10	≫10	ND
**5m**	≫10	≫10	ND
**5n**	9.6 ± 1.2	≫10	ND
**5o**	≫10	≫10	ND
**5p**	≫10	≫10	ND
**5q**	≫10	≫10	ND
**5r**	9.6 ± 0.9	≫10	ND
**5-FU**	4.6 ± 0.2	30 ± 2.5	NA ^2^

^1^ No considerable cytotoxicity was determined; ^2^ Cells were not treated with 5-FU

## Data Availability

Data is contained within the article and supplementary materials.

## Supplementary Materials

The following are available online at, Figures S1–S36: ^1^H-NMR, ^13^C-NMR for **4** & **5a**–**r**; Figures S37–S47: Mass spectra of **5a**, **5b**, **5c**, **5d**, **5e**, **5f**, **5g**, **5j**, **5l**, **5n**, **5q**; Figures S48–S57: Molecular docking figures of **5b**, **5c**, **5d**, **5e**, **5g**, **5k**, **5l**, **5m**, **5o**, **5q**.

## Sample Availability

Samples of the compounds **5a**–**r** are available from the authors.
