# Supplementary material for: Synthesis and Rational Design of New Appended 1,2,3-Triazole-uracil Ensembles as Promising Anti-Tumor Agents via In Silico VEGFR-2 Transferase Inhibition
Source: Molecules. 2021 Mar 30;26(7):1952. doi: 10.3390/molecules26071952 (PMC8037033; doi:10.3390/molecules26071952)
Supplement: Supplementary file 1 [file molecules-26-01952-s001.pdf]

molecules

Supplementary files

Synthesis and Rational Design of New Appended 1,2,3-Triazole-Uracil Ensembles as Promising Anti-Tumor Agents via In Silico VEGFR-2 Transferase Inhibition

Nadipolla Naresh Reddy [1,†], Sung-Jen Hung [2,3,†], Merugu Kumara Swamy [1], Anathula Sanjeev [1], Vankadari Srinivasa Rao [1], Rondla Rohini [1], Atcha Krishnam Raju [4], Kuthati Bhaskar [1], Anren Hu [3,5,*] and Puchakayala Muralidhar Reddy [1,*]

1 Department of Chemistry, Osmania University, Hyderabad, Telangana 500007, India; nareshnadipolla@gmail.com (N.N.R.); kumaraswamy.sci@gmail.com (M.K.S.); sanjeev.ku610@gmail.com (A.S.) chemsrinu44@gmail.com (V.S.R.); prmnreddy@osmania.ac.in (R.R.); kuthati18@osmania.ac.in (K.B.)
2 Department of Dermatology, Buddhist Tzu-Chi General Hospital, Hualien 97002, Taiwan; md.hong@msa.hinet.net
3 Institute of Medical Sciences, Tzu-Chi University, Hualien 97002, Taiwan; anren@gms.tcu.edu.tw
4 Department of Chemistry, Nizam College, Osmania University, Hyderabad 500001, TS, India; krishnamrajua@osmania.ac.in
5 Department of Laboratory Medicine and Biotechnology, College of Medicine, Tzu-Chi University, Hualien, Taiwan; anren@gms.tcu.edu.tw
† These authors contributed equally to this work.
* Correspondence: pmdreddy@osmania.ac.in or pmdreddy@gmail.com (P.M.R.); anren@gms.tcu.edu.tw (A.H.); Tel.: +91-9848792423 (P.M.R.); +886-3-8565301 (ext. 2334 or 2335) (A.H.); Fax: +886-3-8571917 (A.H.)

^{1}H-and ^{13}C-NMR spectra were recorded using Bruker Avance II 400 and 100 MHz spectrometers in 99.99% DMSO-d_6 and 99.82% $CDCl_3$ (2 mg/mL) using TMS as the standard solvent. ESI-MS spectra were measured on LCMS 2010 VG mass spectrometer in Methanol/DCM solvent (10 µg/mL).

^{1}H-NMR for **4**; ^{1}H-NMR & ^{13}C-NMR for **5a–r**; ESI-Mass for 5a, 5b, 5c, 5d, 5e, 5f, 5g, 5j, 5l, 5n and 5q; Docking figures of 5b, 5c, 5d, 5e, 5g, 5k, 5l, 5m, 5o and 5q

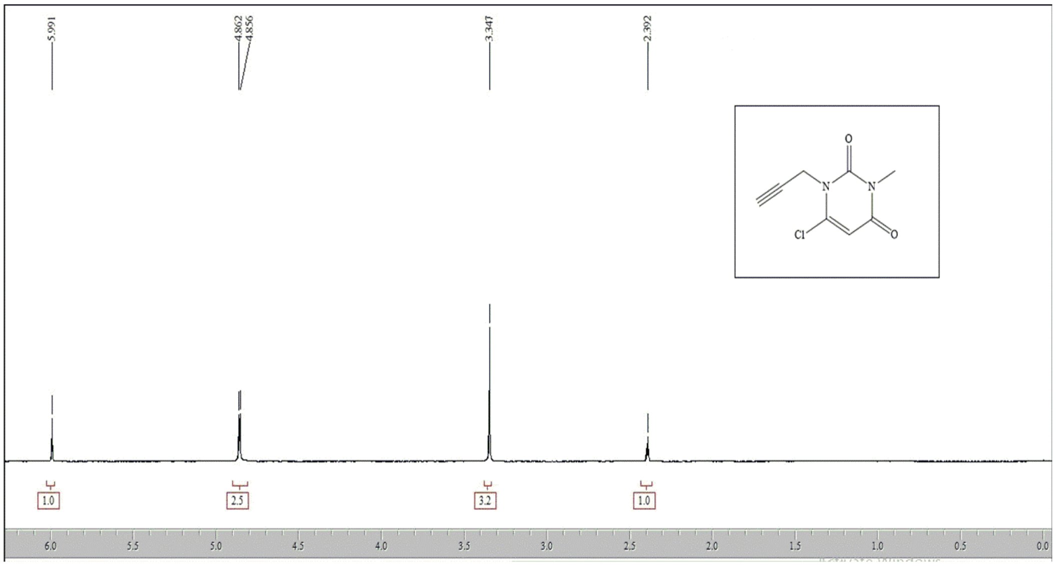

Figure S1. ^{1}H-NMR of 6-chloro-3-methyl-1-(prop-2-yn-1-yl)pyrimidine-2,4(1H,3H)-dione (**4**).

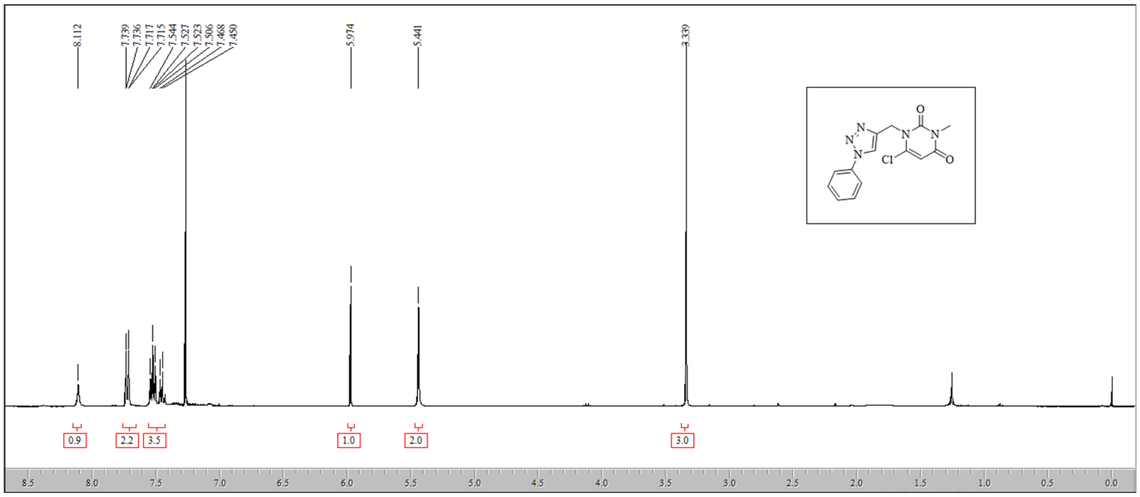

Figure S2. ^{1}H-NMR 6-chloro-3-methyl-1-((1-phenyl-1H-1,2,3-triazol-4-yl)methyl)pyrimidine-2,4(1H,3H)-dione (**5a**).

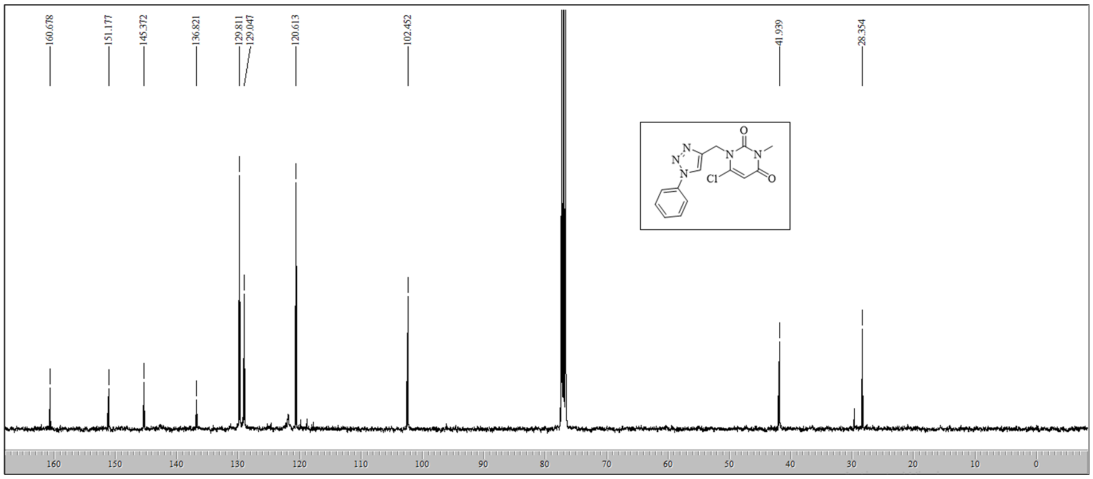

Figure S3. ^{13}C-NMR 6-chloro-3-methyl-1-((1-phenyl-1H-1,2,3-triazol-4-yl)methyl)pyrimidine-2,4(1H,3H)-dione (**5a**).

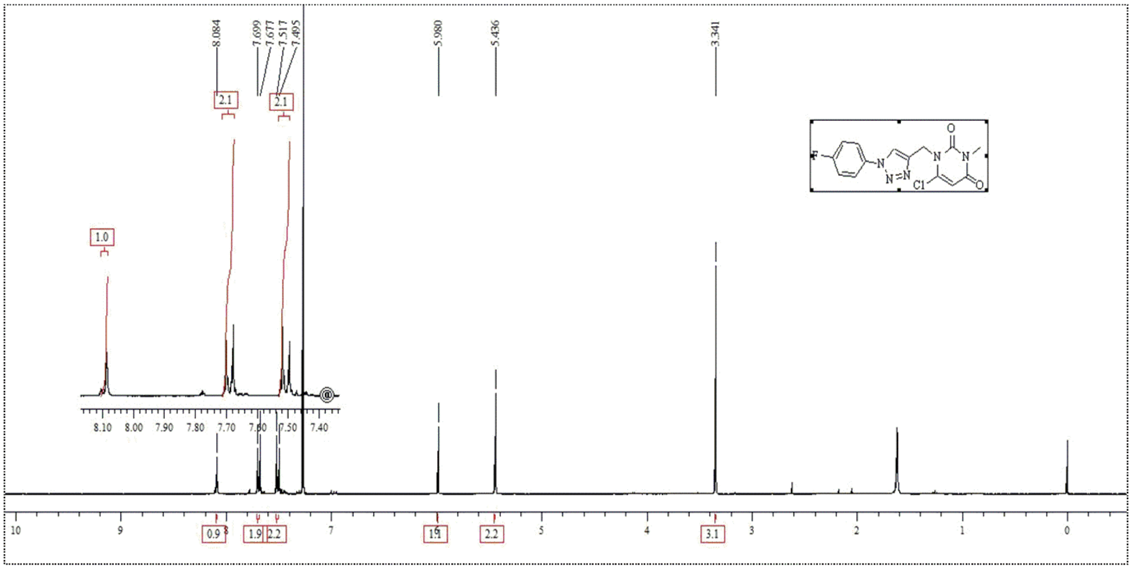

Figure S4. ^{1}H-NMR of 6-chloro-1-((1-(4-fluorophenyl)-1H-1,2,3-triazol-4-yl)methyl)-3-methylpyrimidine-2,4(1H,3H)-dione (**5b**).

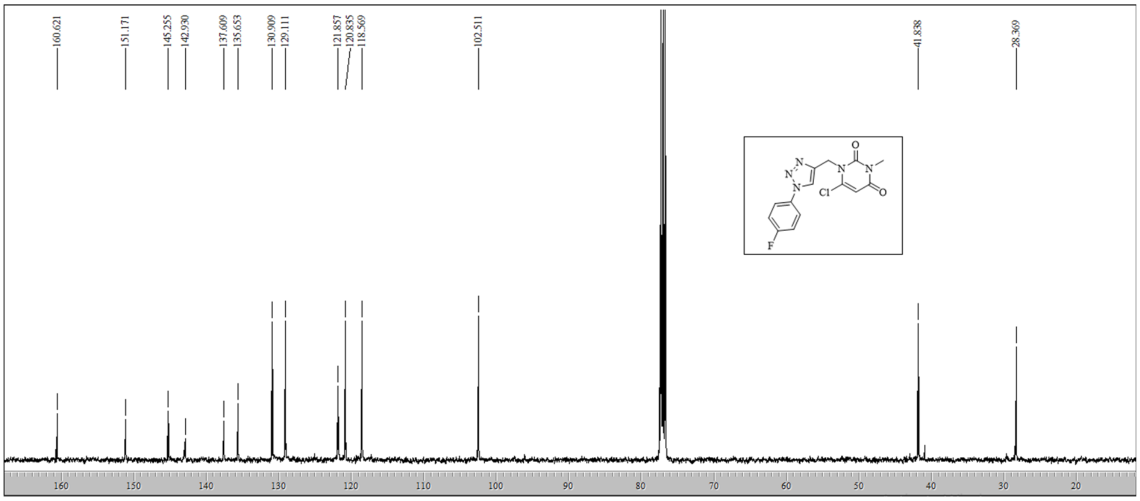

Figure S5. ^{13}C-NMR 6-chloro-1-((1-(4-fluorophenyl)-1H-1,2,3-triazol-4-yl)methyl)-3-methylpyrimidine-2,4(1H,3H)-dione (**5b**).

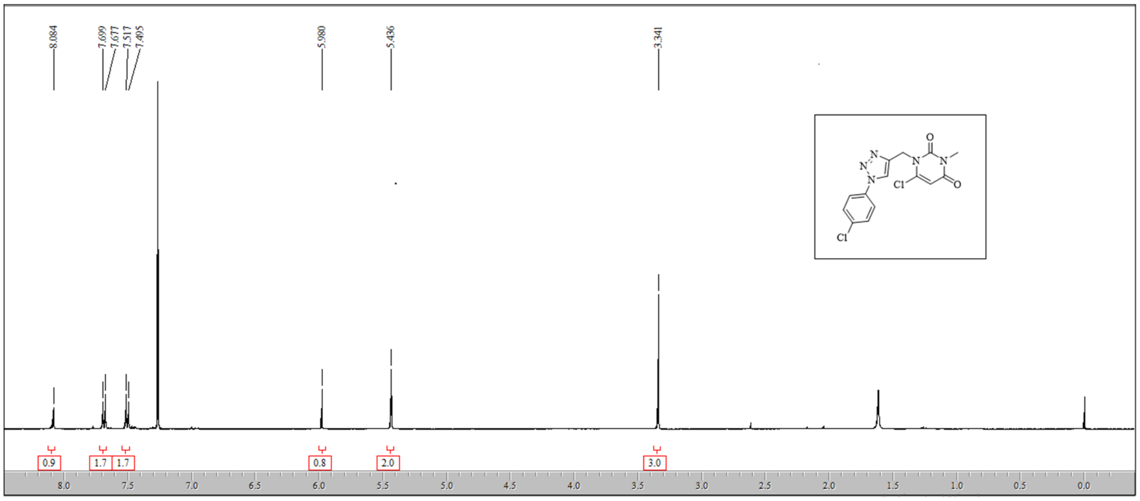

Figure S6. ^{1}H-NMR of 6-chloro-1-((1-(4-chlorophenyl)-1H-1,2,3-triazol-4-yl)methyl)-3-methylpyrimidine-2,4(1H,3H)-dione (**5c**).

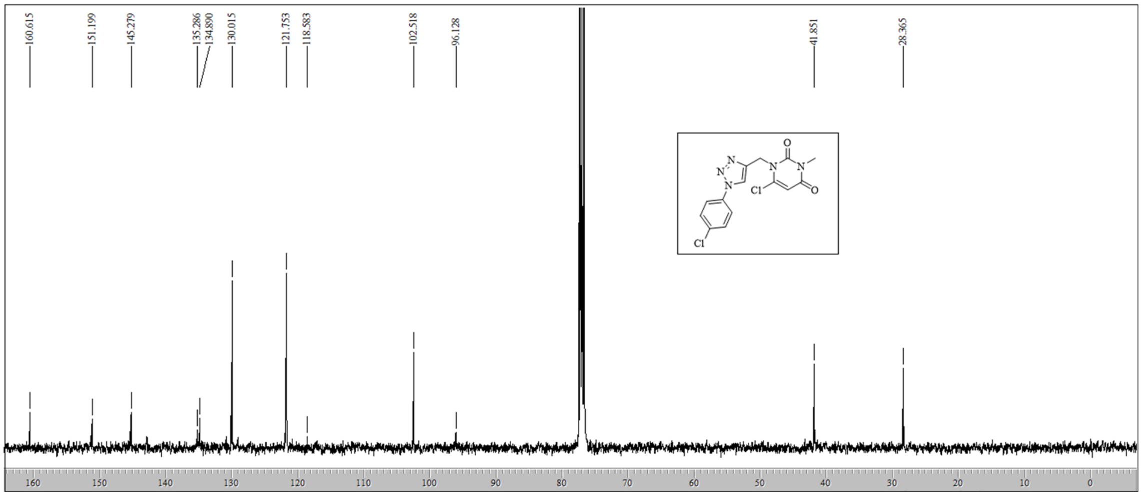

Figure S7. ^{13}C-NMR of 6-chloro-1-((1-(4-chlorophenyl)-1H-1,2,3-triazol-4-yl)methyl)-3-methylpyrimidine-2,4(1H,3H)-dione (**5c**).

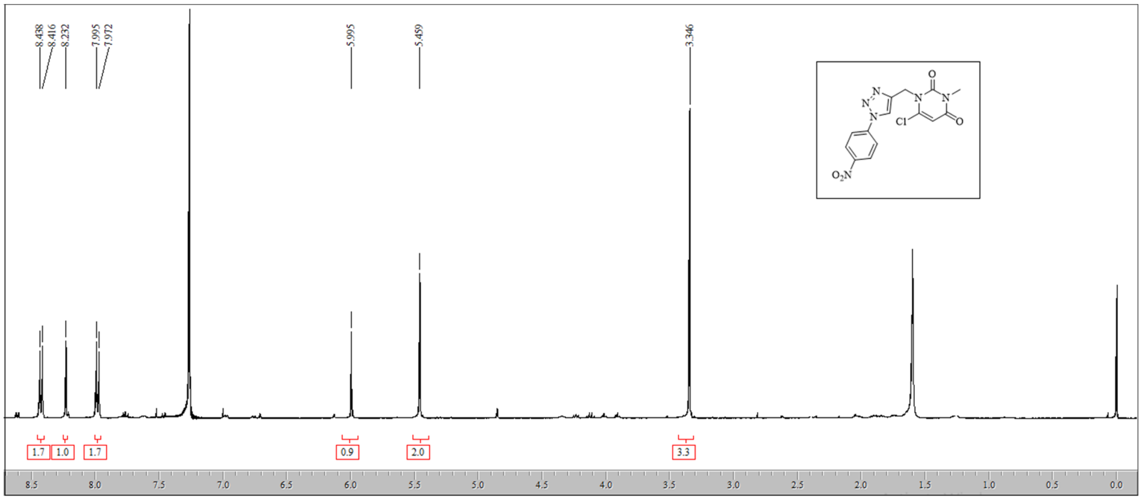

Figure S8. ^{1}H-NMR of 6-chloro-3-methyl-1-((1-(4-nitrophenyl)-1H-1,2,3-triazol-4-yl)methyl)pyrimidine-2,4(1H,3H)-dione (**5d**).

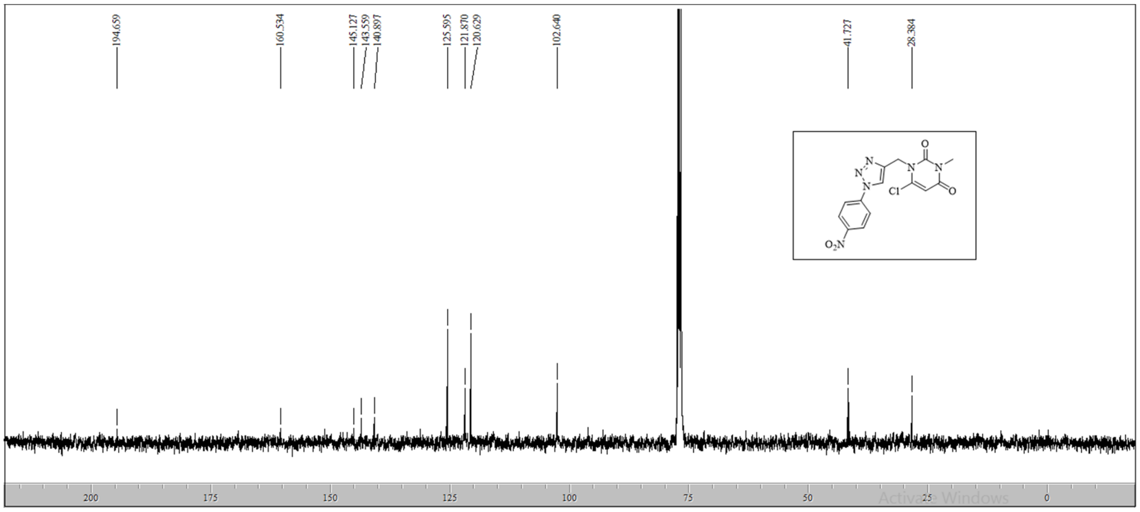

Figure S9. ^{13}C-NMR of 6-chloro-3-methyl-1-((1-(4-nitrophenyl)-1H-1,2,3-triazol-4-yl)methyl)pyrimidine-2,4(1H,3H)-dione (**5d**).

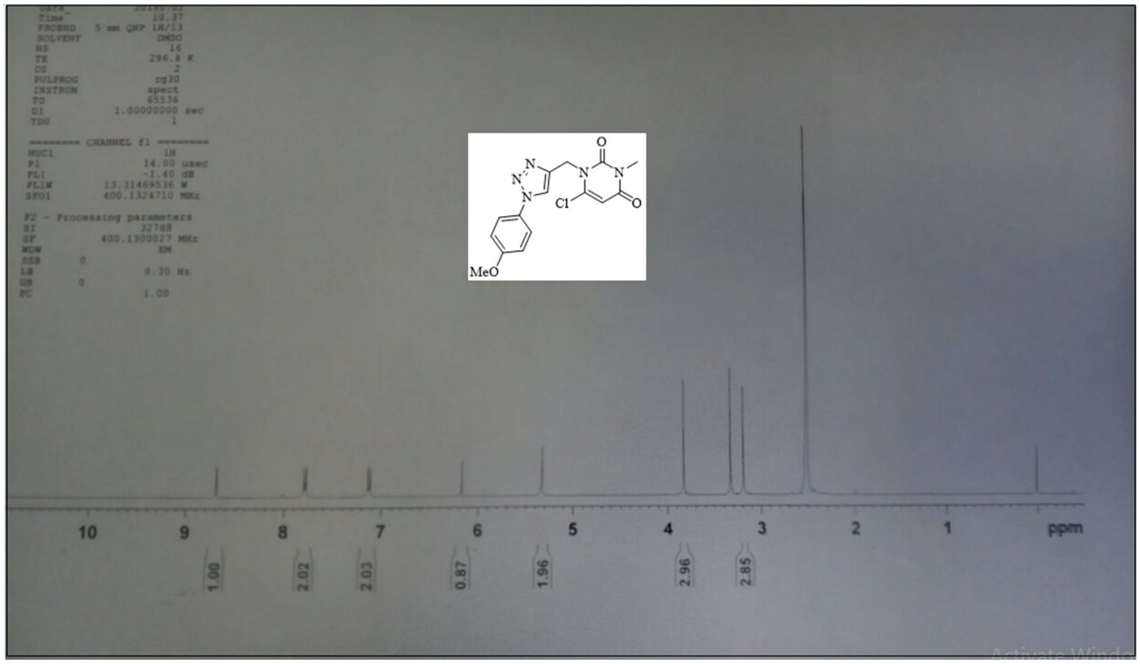

Figure S10. ^{1}H-NMR of 6-chloro-1-((1-(4-methoxyphenyl)-1H-1,2,3-triazol-4-yl)methyl)-3-methylpyrimidine-2,4(1H,3H)-dione (**5e**).

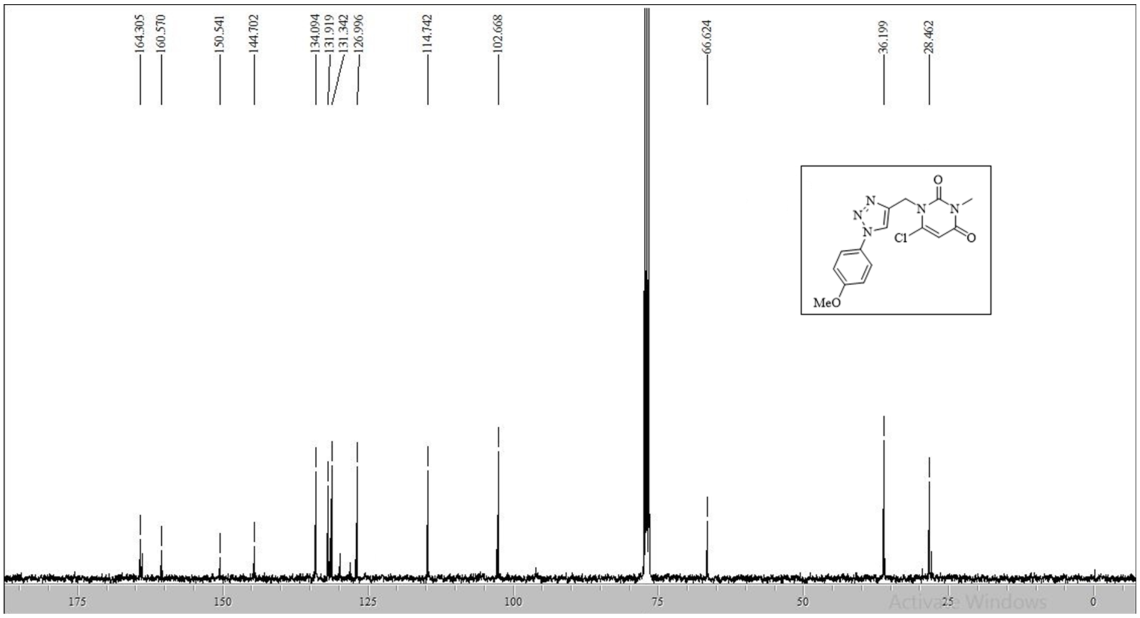

Figure S11. ^{13}C-NMR of 6-chloro-1-((1-(4-methoxyphenyl)-1H-1,2,3-triazol-4-yl)methyl)-3-methylpyrimidine-2,4(1H,3H)-dione (**5e**).

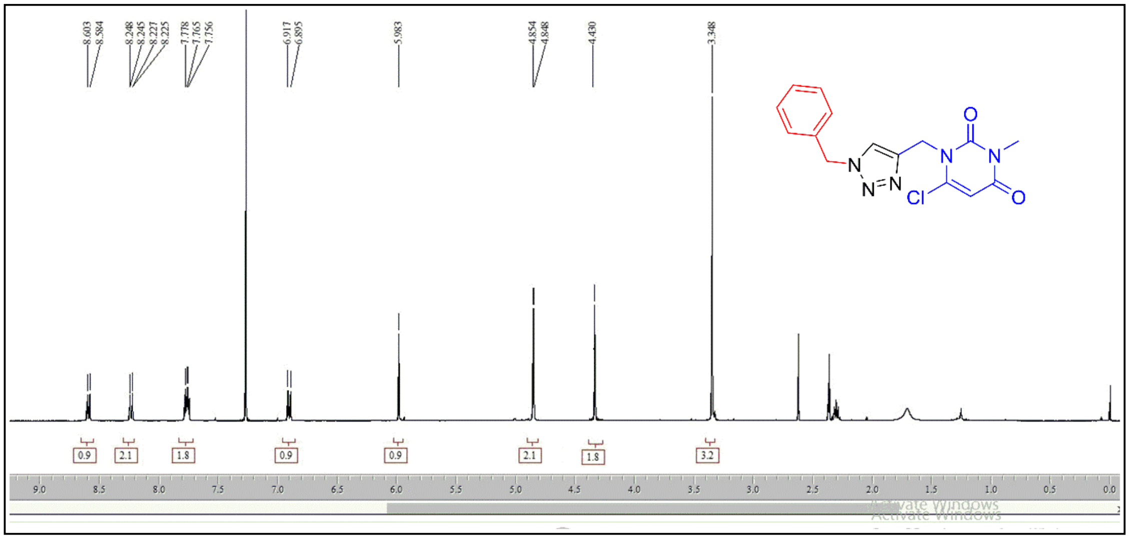

Figure S12. ^{1}H-NMR of 1-((1-benzyl-1H-1,2,3-triazol-4-yl)methyl)-6-chloro-3-methylpyrimidine-2,4(1H,3H)-dione (**5f**).

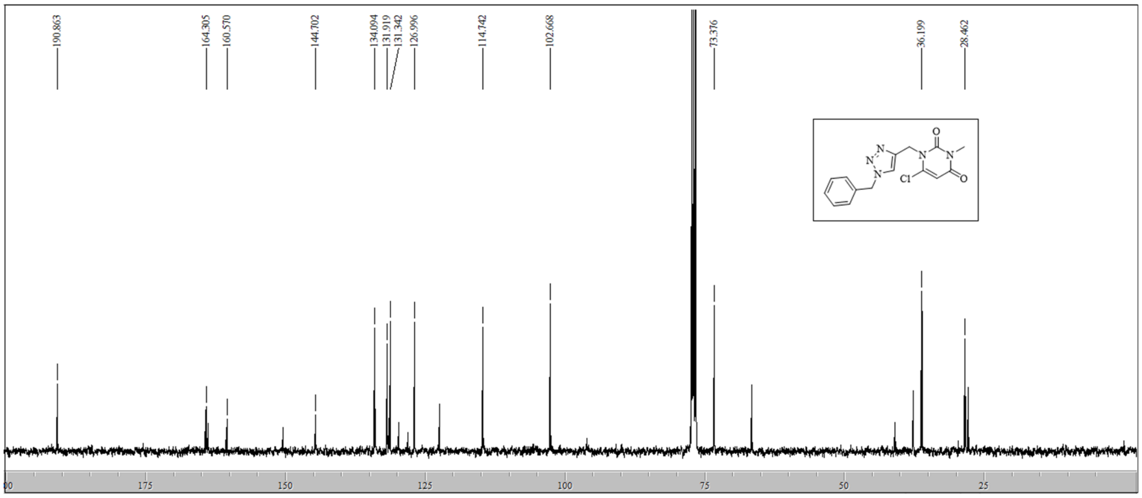

Figure 13. ^{13}C-NMR of 1-((1-benzyl-1H-1,2,3-triazol-4-yl)methyl)-6-chloro-3-methylpyrimidine-2,4(1H,3H)-dione (**5f**).

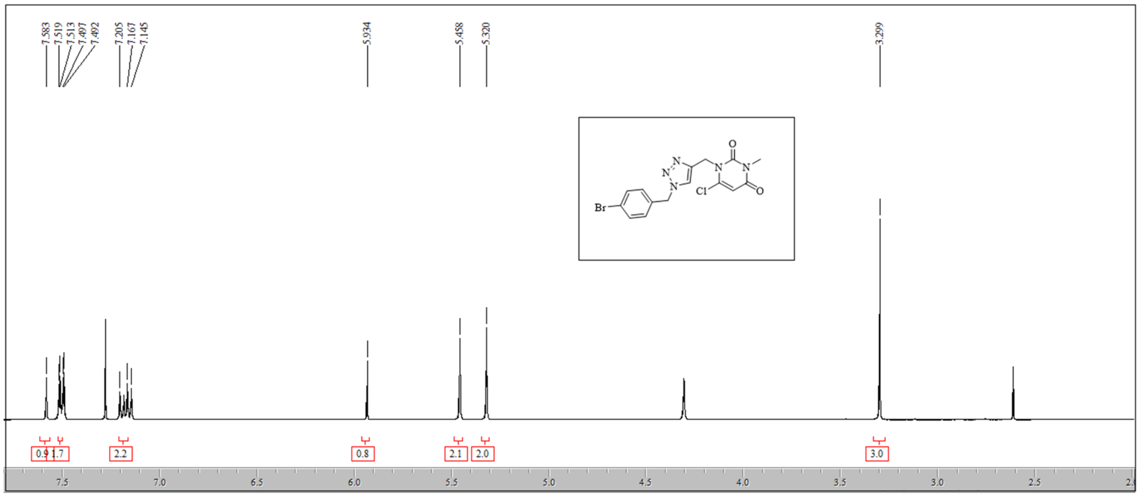

Figure S14. ^{1}H-NMR of 1-((1-(4-bromobenzyl)-1H-1,2,3-triazol-4-yl)methyl)-6-chloro-3-methylpyrimidine-2,4(1H,3H)-dione (**5g**).

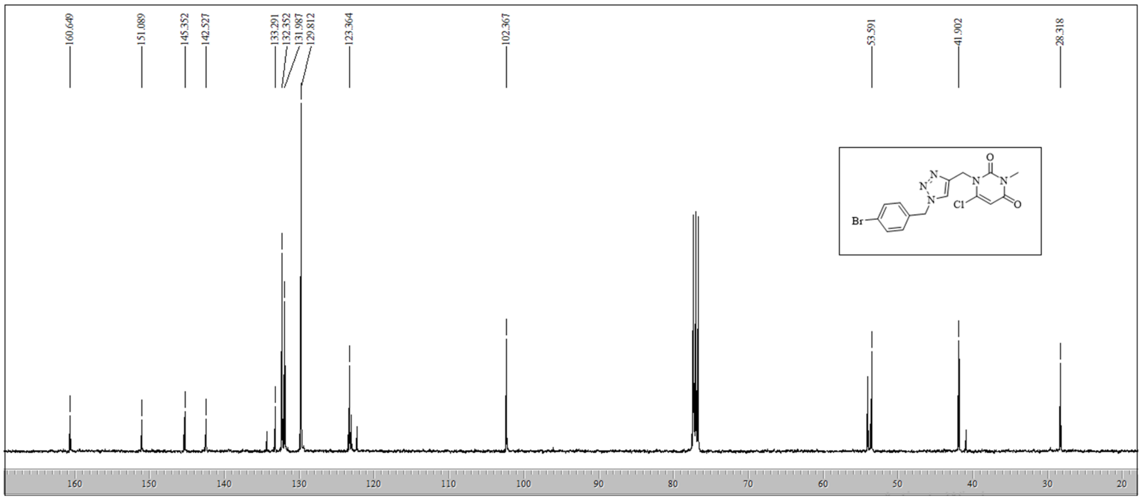

Figure S15. ^{13}C-NMR of 1-((1-(4-bromobenzyl)-1H-1,2,3-triazol-4-yl)methyl)-6-chloro-3-methylpyrimidine-2,4(1H,3H)-dione (**5g**).

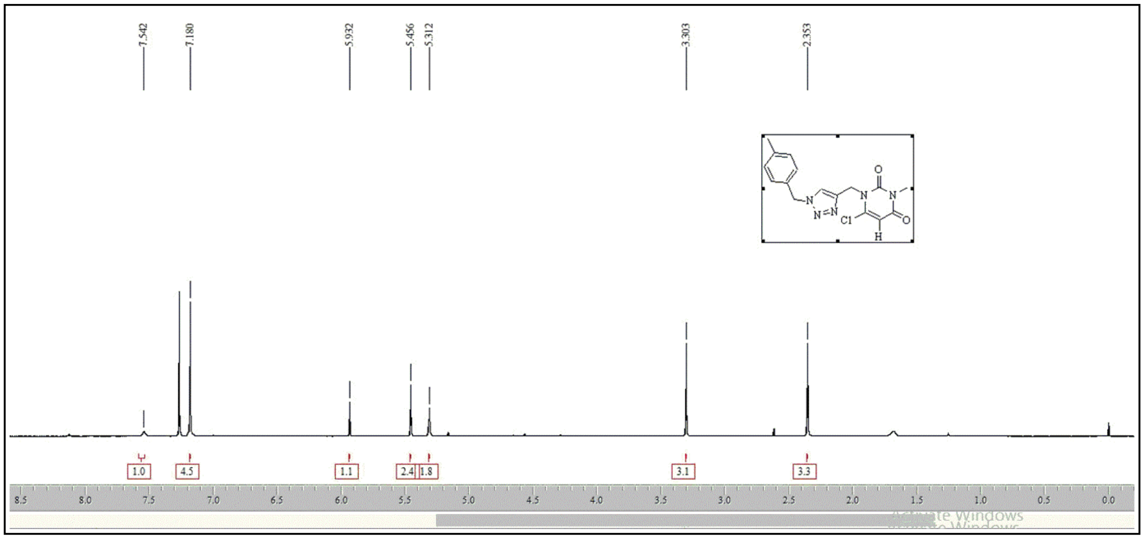

Figure S16. ^{1}H-NMR of 6-chloro-3-methyl-1-((1-(4-methylbenzyl)-1H-1,2,3-triazol-4-yl)methyl)pyrimidine-2,4(1H,3H)-dione (**5h**).

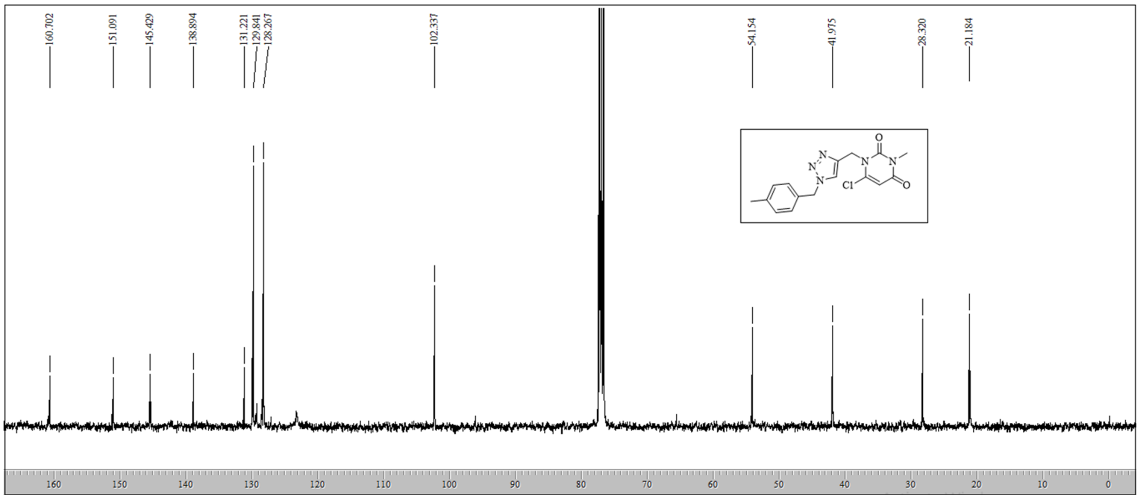

Figure 17. ^{13}C-NMR of 6-chloro-3-methyl-1-((1-(4-methylbenzyl)-1H-1,2,3-triazol-4-yl)methyl)pyrimidine-2,4(1H,3H)-dione (**5h**).

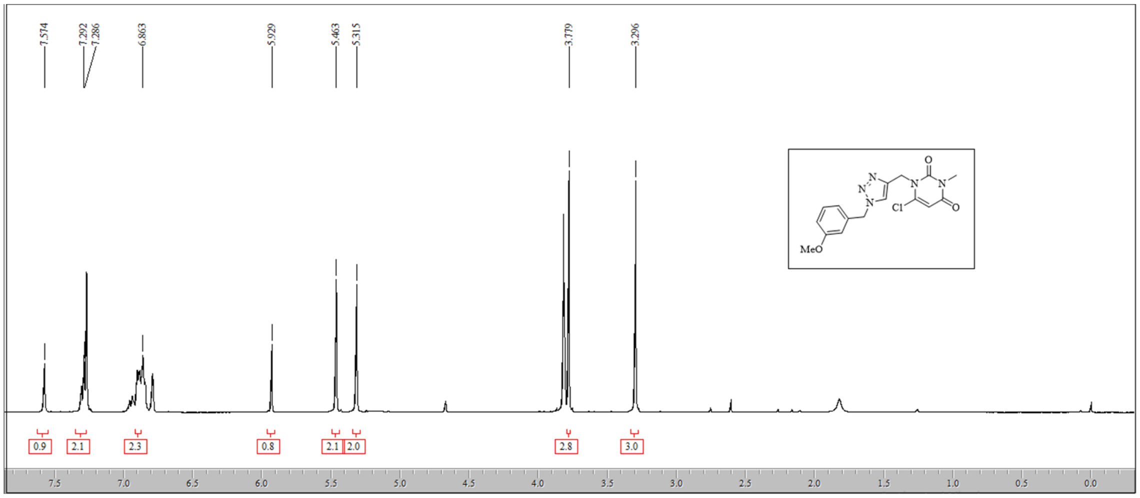

Figure S18. ¹H-NMR of 6-chloro-1-((1-(3-methoxybenzyl)-1H-1,2,3-triazol-4-yl)methyl)-3-methylpyrimidine-2,4(1H,3H)-dione (**5i**).

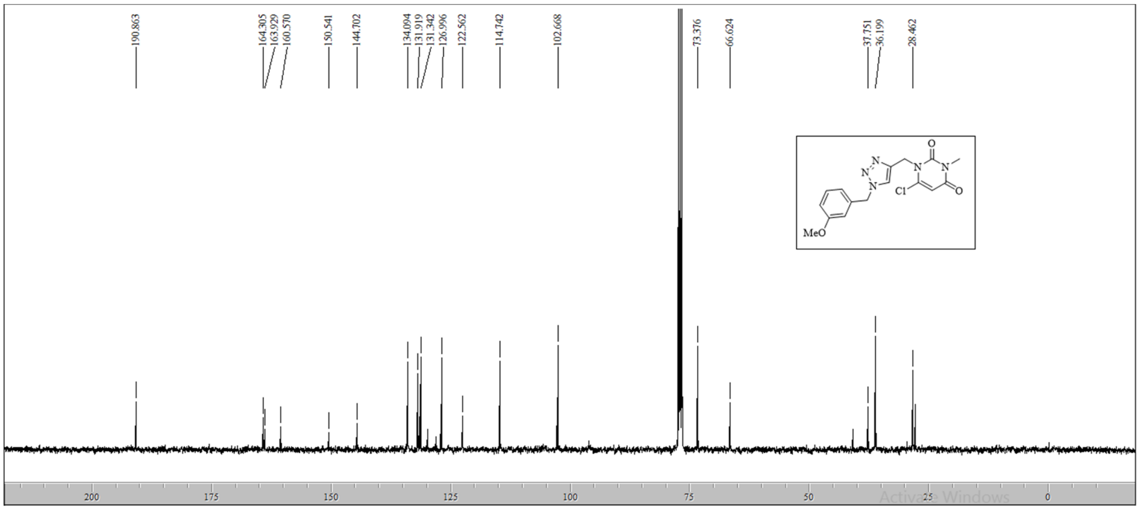

Figure S19. ¹³C-NMR of 6-chloro-1-((1-(3-methoxybenzyl)-1H-1,2,3-triazol-4-yl)methyl)-3-methylpyrimidine-2,4(1H,3H)-dione (**5i**).

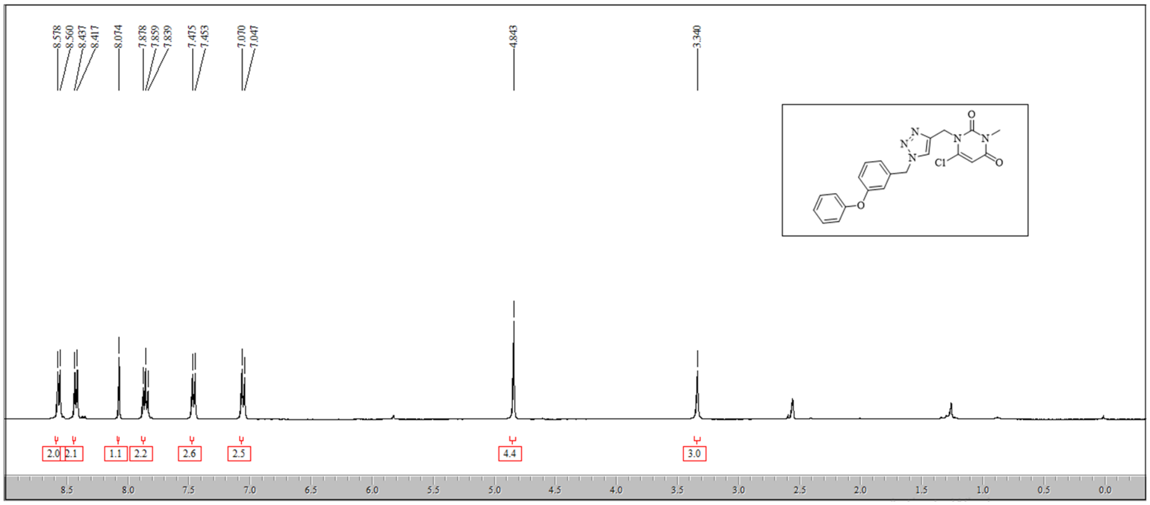

Figure S20. ^{1}H-NMR of 6-chloro-3-methyl-1-((1-(3-phenoxybenzyl)-1H-1,2,3-triazol-4-yl)methyl)pyrimidine-2,4(1H,3H)-dione (**5j**).

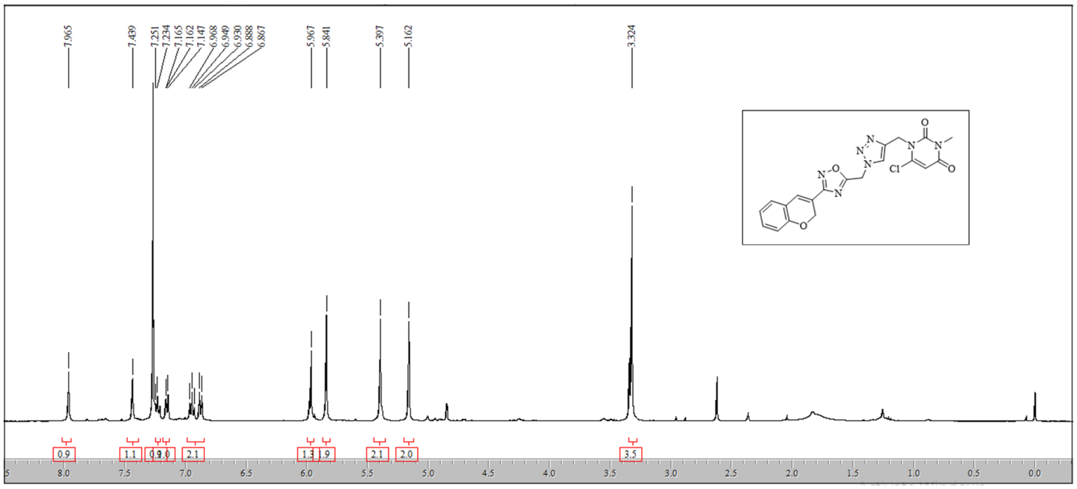

Figure S21. ^{1}H-NMR of 1-((1-((3-(2H-chromen-3-yl)-1,2,4-oxadiazol-5-yl)methyl)-1H-1,2,3-triazol-4-yl)methyl)-6-chloro-3-methylpyrimidine-2,4(1H,3H)-dione (**5k**).

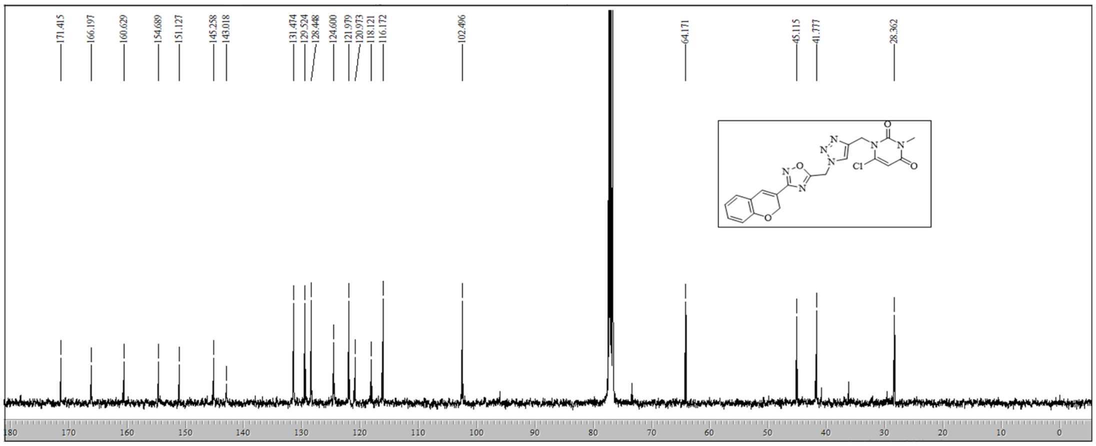

Figure S22. ^{13}C-NMR of 1-((1-((3-(2H-chromen-3-yl)-1,2,4-oxadiazol-5-yl)methyl)-1H-1,2,3-triazol-4-yl)methyl)-6-chloro-3-methylpyrimidine-2,4(1H,3H)-dione (**5k**).

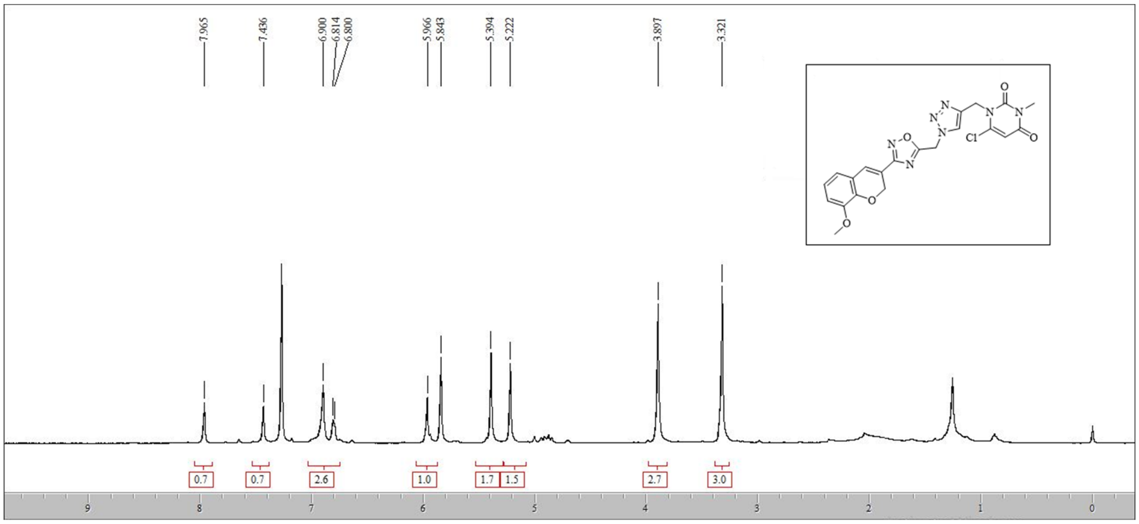

Figure S23. ^{1}H-NMR of 6-chloro-1-((1-((3-(8-methoxy-2H-chromen-3-yl)-1,2,4-oxadiazol-5-yl)methyl)-1H-1,2,3-triazol-4-yl)methyl)-3-methylpyrimidine-2,4(1H,3H)-dione (**5l**).

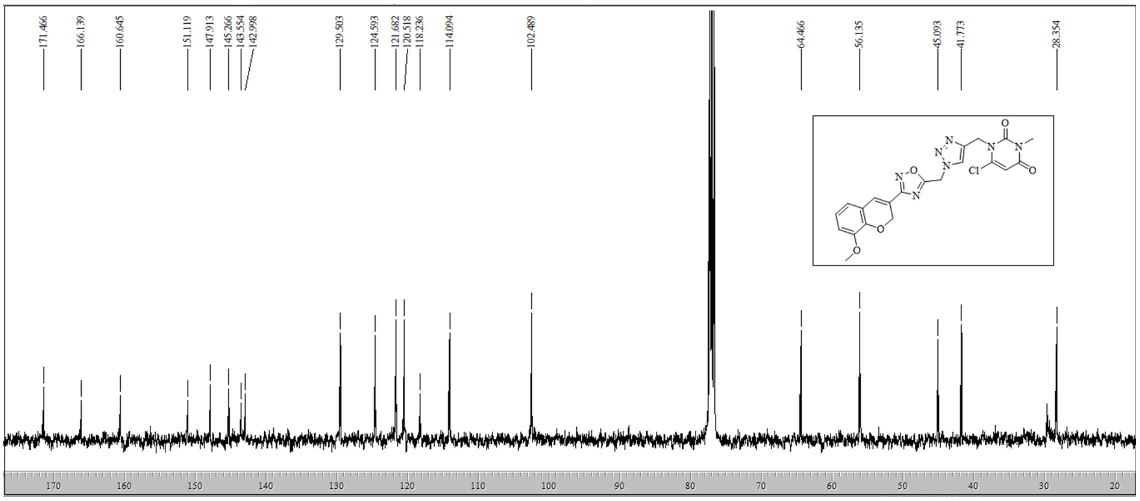

Figure S24. ^{13}C NMR of 6-chloro-1-((1-((3-(8-methoxy-2H-chromen-3-yl)-1,2,4-oxadiazol-5-yl)methyl)-1H-1,2,3-triazol-4-yl)methyl)-3-methylpyrimidine-2,4(1H,3H)-dione (**5l**).

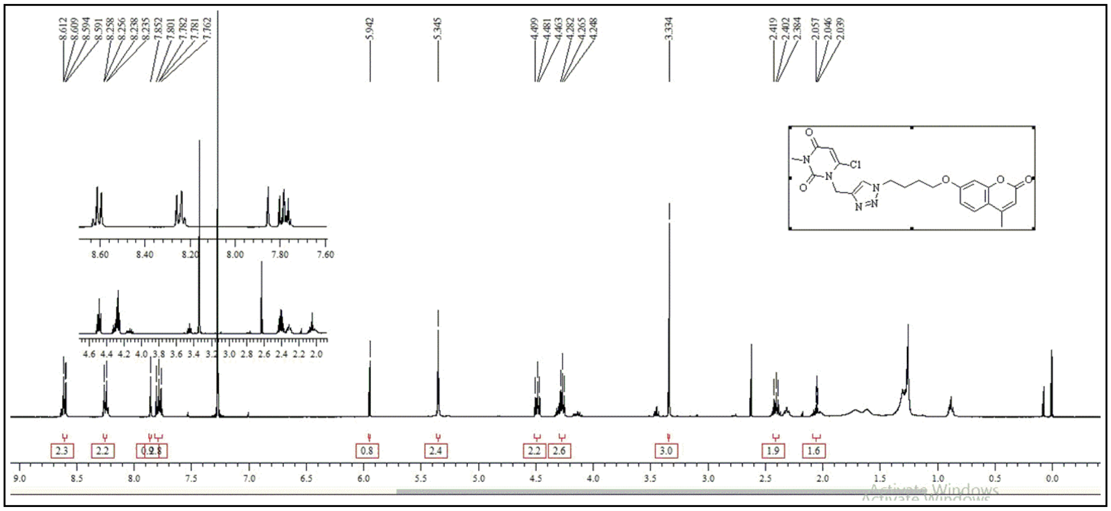

Figure S25. ^{1}H-NMR of 6-chloro-3-methyl-1-((1-(4-((4-methyl-2-oxo-2H-chromen-7-yl)oxy)butyl)-1H-1,2,3-triazol-4-yl)methyl)pyrimidine-2,4(1H,3H)-dione (**5m**).

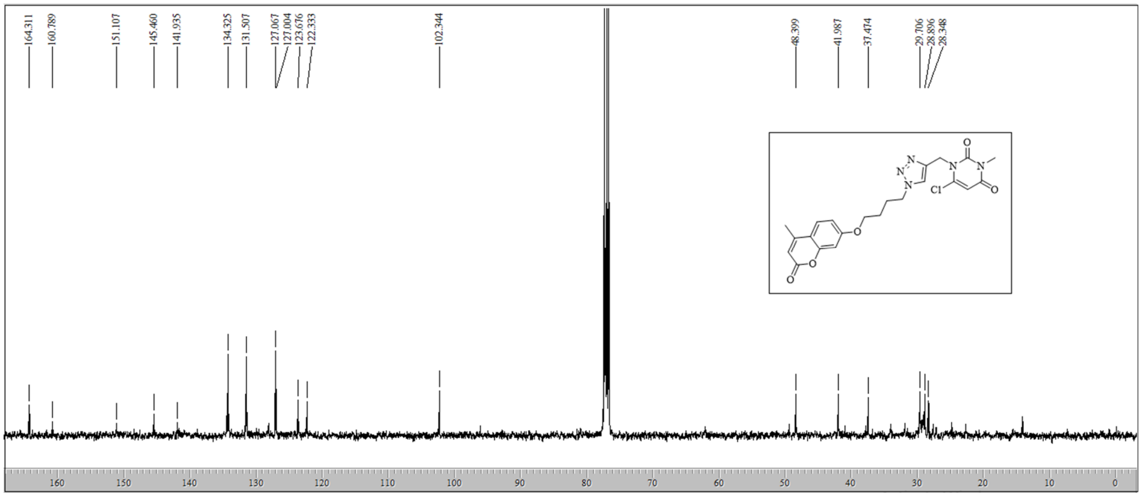

Figure S26. ^{13}C-NMR of 6-chloro-3-methyl-1-((1-(4-((4-methyl-2-oxo-2H-chromen-7-yl)oxy)butyl)-1H-1,2,3-triazol-4-yl)methyl)pyrimidine-2,4(1H,3H)-dione (**5m**).

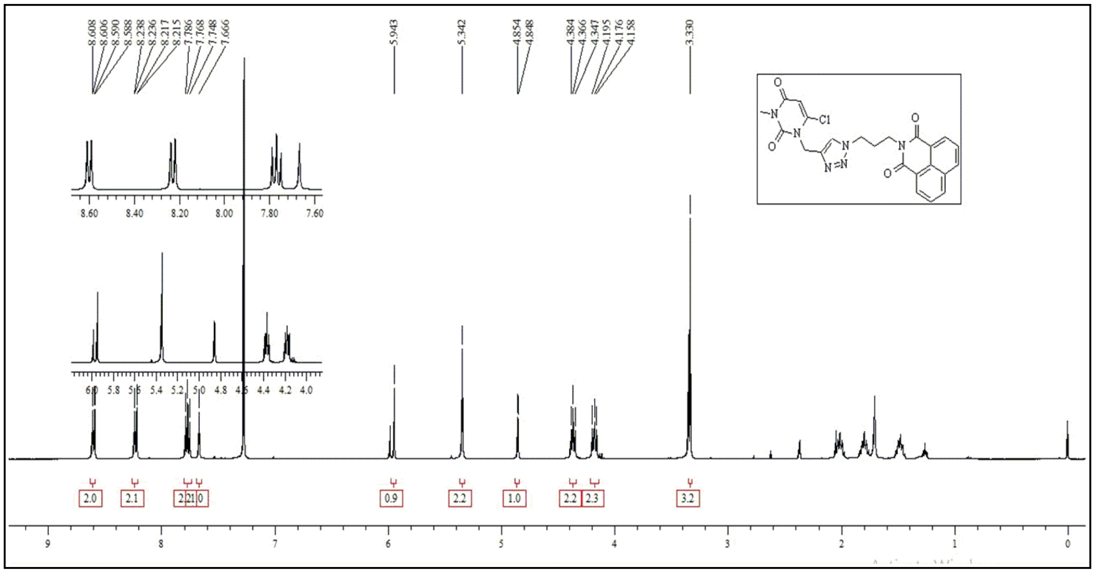

Figure S27. ^{1}H-NMR of 2-(3-(4-((6-chloro-3-methyl-2,4-dioxo-3,4-dihydropyrimidin-1(2H)-yl)methyl)-1H-1,2,3-triazol-1-yl)propyl)-1H-benzo[ω]isoquinoline-1,3(2H)-dione (**5n**).

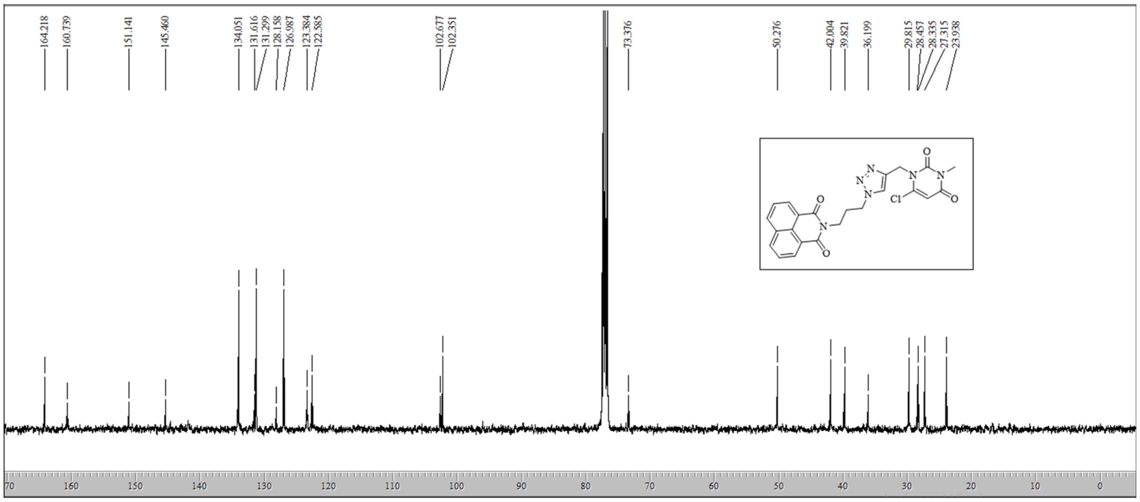

Figure S28. ^{13}C-NMR of 2-(3-(4-((6-chloro-3-methyl-2,4-dioxo-3,4-dihydropyrimidin-1(2H)-yl)methyl)-1H-1,2,3-triazol-1-yl)propyl)-1H-benzo[ω]isoquinoline-1,3(2H)-dione (**5n**).

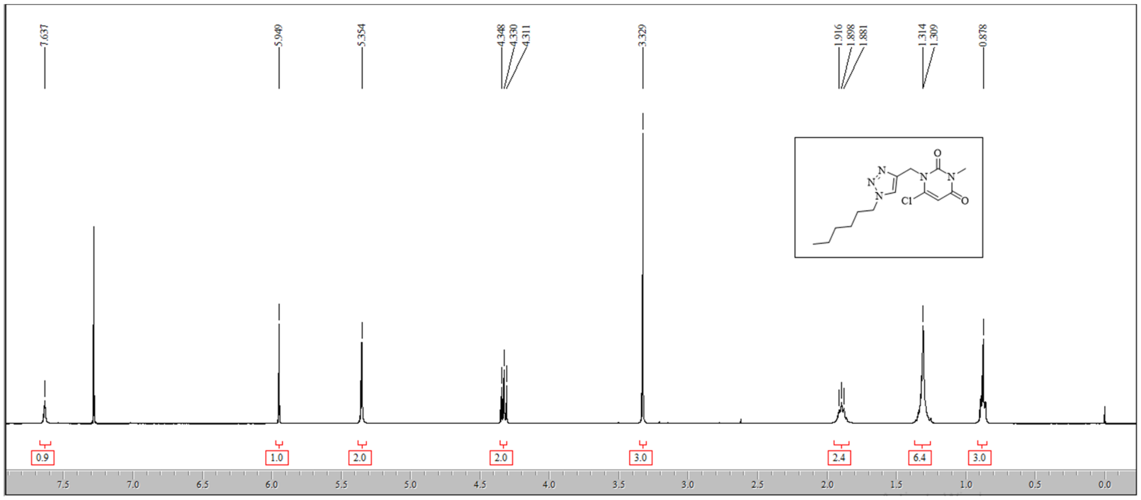

Figure S29. ^{1}H-NMR of 6-chloro-1-((1-hexyl-1H-1,2,3-triazol-4-yl)methyl)-3-methylpyrimidine-2,4(1H,3H)-dione (**5o**).

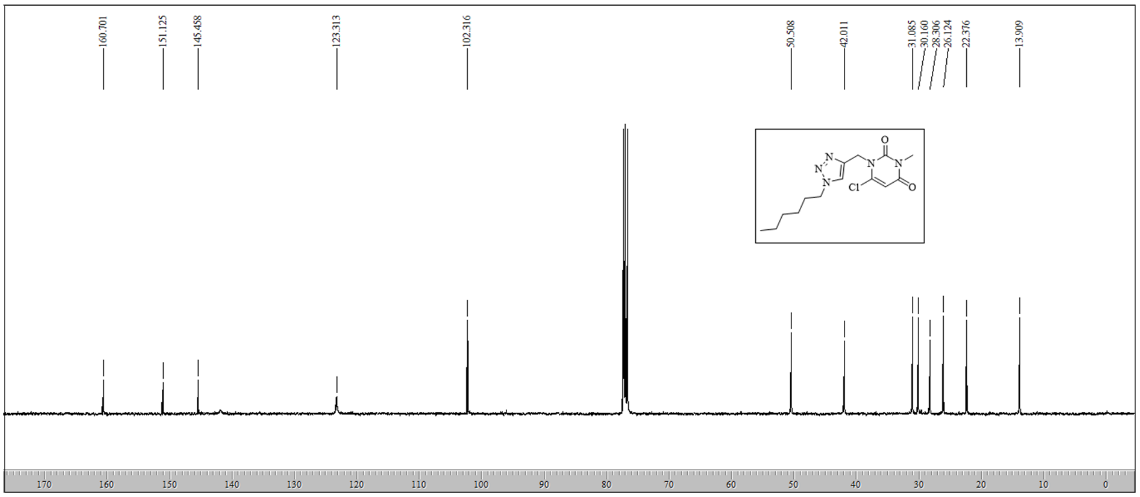

Figure S30. ^{13}C-NMR of 6-chloro-1-((1-hexyl-1H-1,2,3-triazol-4-yl)methyl)-3-methylpyrimidine-2,4(1H,3H)-dione (**5o**).

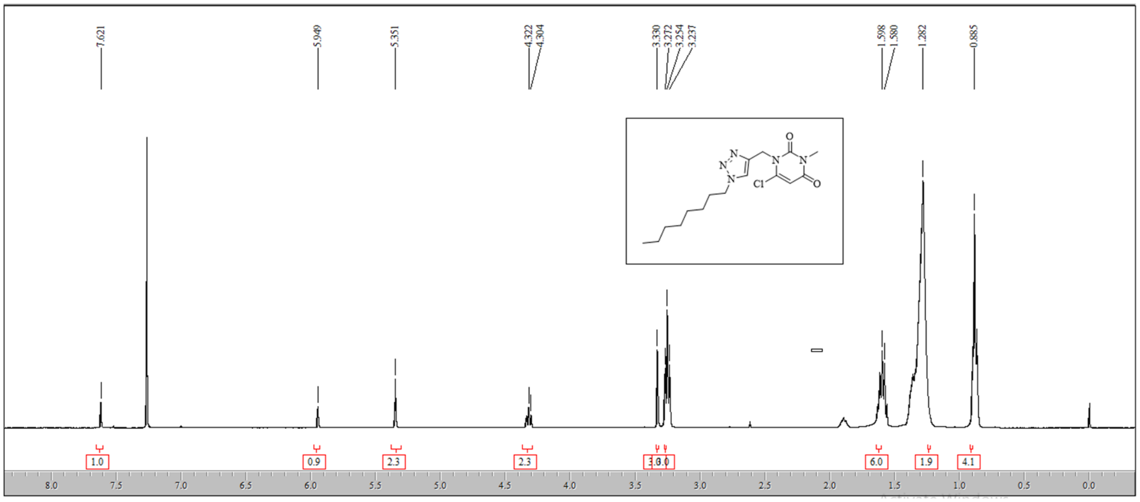

Figure S31. ^{1}H-NMR of 6-chloro-3-methyl-1-((1-octyl-1H-1,2,3-triazol-4-yl)methyl)pyrimidine-2,4(1H,3H)-dione (**5p**).

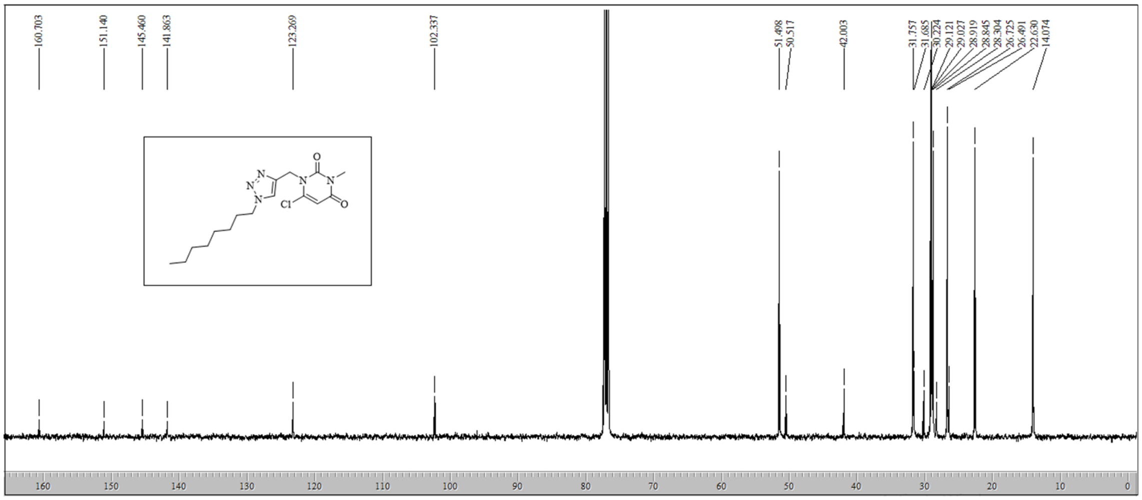

Figure S32. ^{13}C-NMR of 6-chloro-3-methyl-1-((1-octyl-1H-1,2,3-triazol-4-yl)methyl)pyrimidine-2,4(1H,3H)-dione (**5p**).

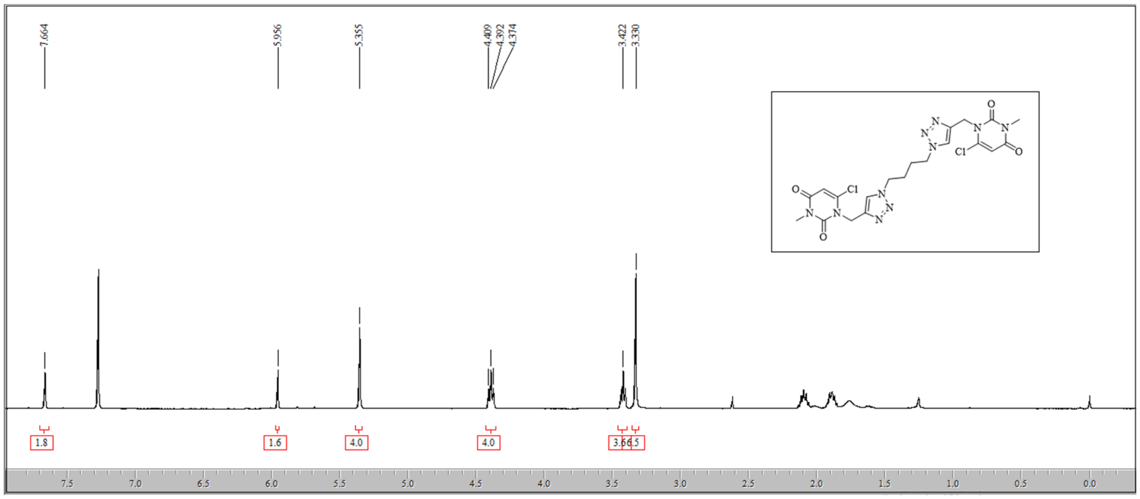

Figure S33. ^{1}H-NMR of 1,1'-((1,1'-(butane-1,4-diyl)bis(1H-1,2,3-triazole-4,1-diyl))bis(methylene))bis(6-chloro-3-methylpyrimidine-2,4(1H,3H)-dione) (**5q**).

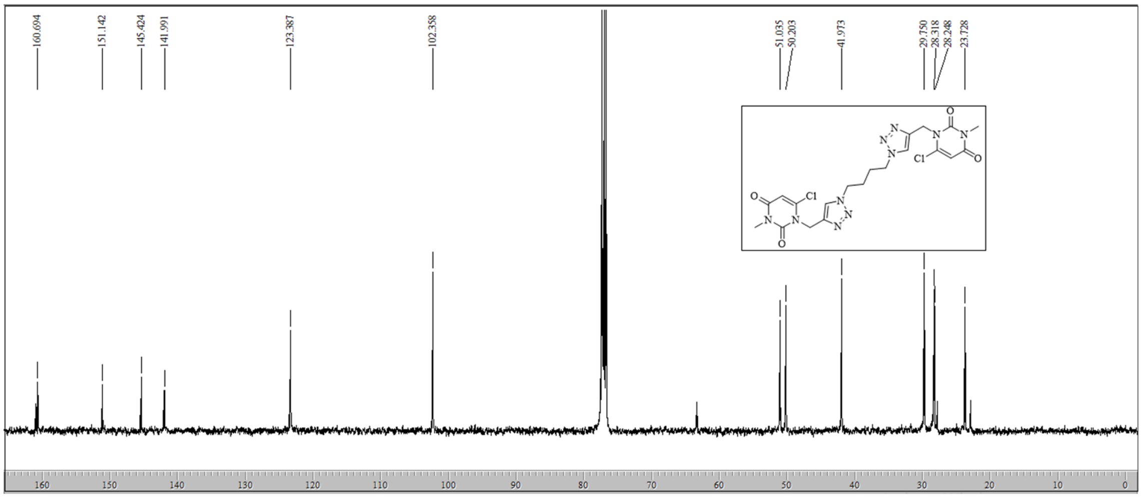

Figure S34. ^{13}C-NMR of 1,1'-((1,1'-(butane-1,4-diyl)bis(1H-1,2,3-triazole-4,1-diyl))bis(methylene))bis(6-chloro-3-methylpyrimidine-2,4(1H,3H)-dione) (**5q**).

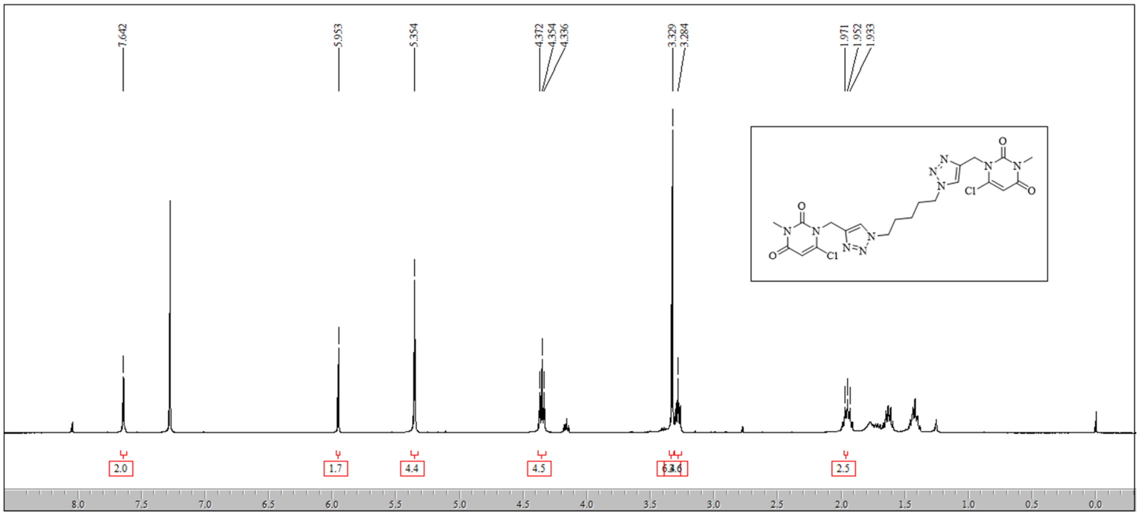

Figure S35. ¹H-NMR of 1,1'-((1,1'-(pentane-1,5-diyl)bis(1H-1,2,3-triazole-4,1-diyl))bis(methylene))bis(6-chloro-3-methylpyrimidine-2,4(1H,3H)-dione) (**5r**).

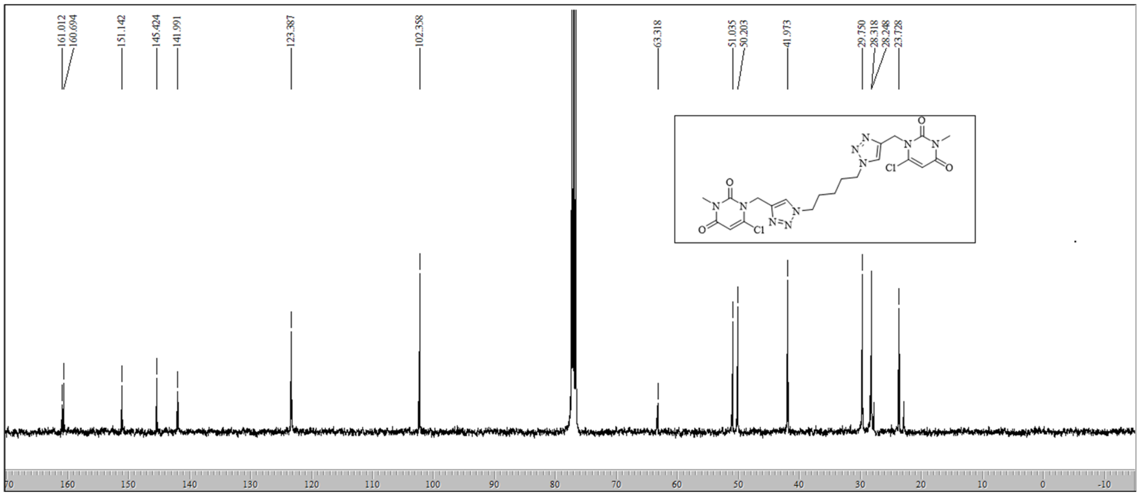

Figure S36. ¹³C-NMR of 1,1'-((1,1'-(pentane-1,5-diyl)bis(1H-1,2,3-triazole-4,1-diyl))bis(methylene))bis(6-chloro-3-methylpyrimidine-2,4(1H,3H)-dione) (**5r**).

ESI-Mass

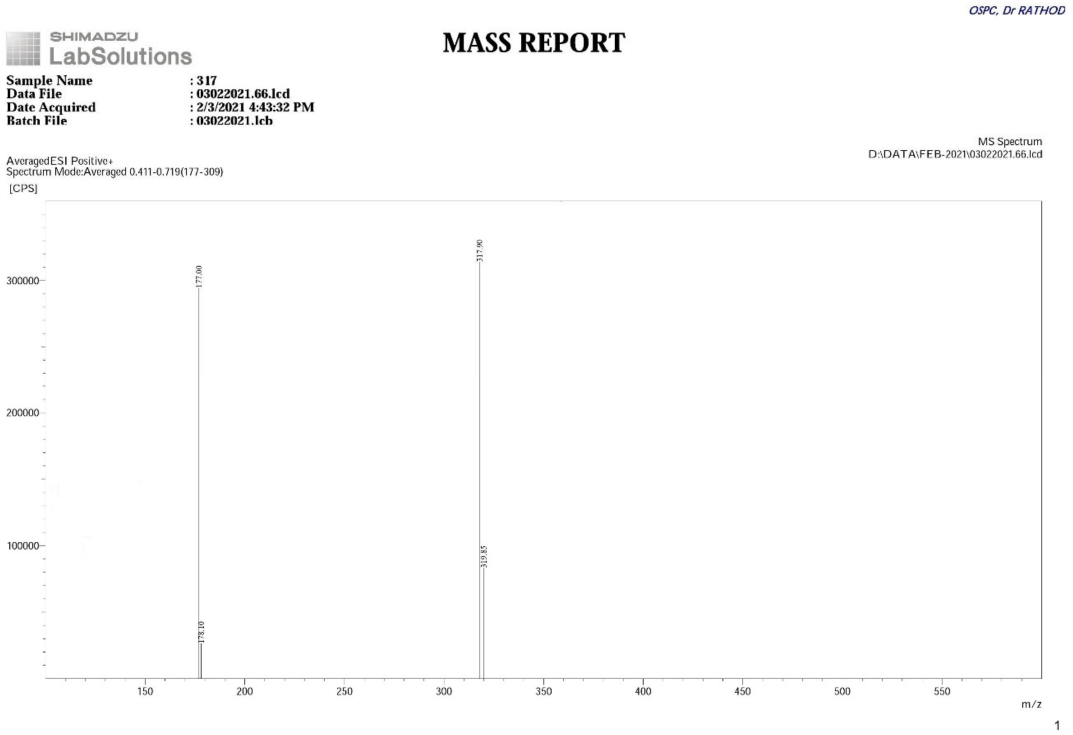

Figure S37. ESI mass spectrum of **5a.**

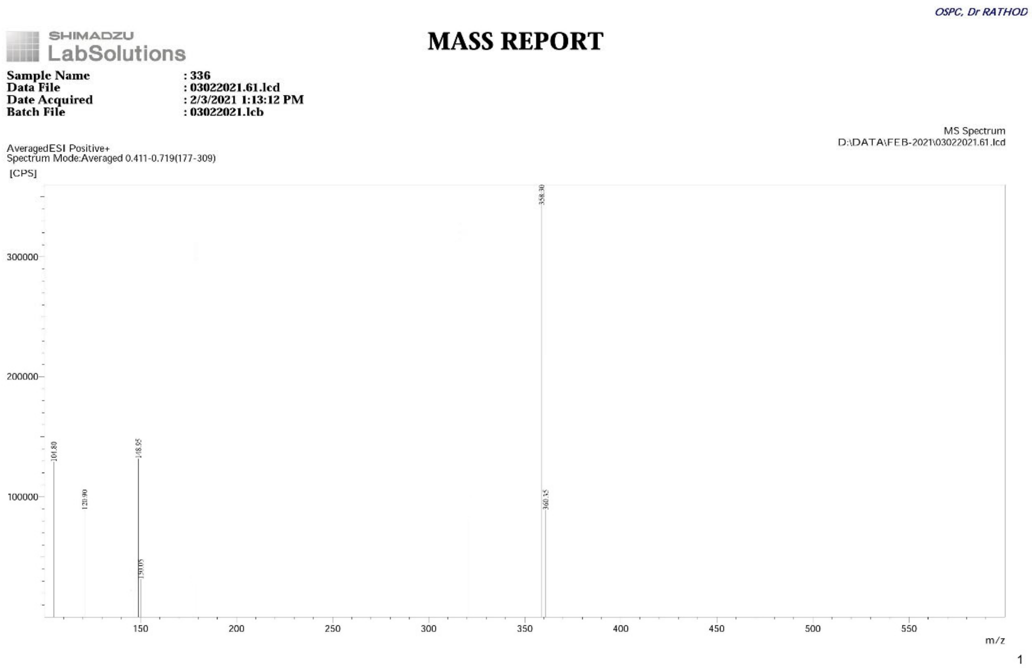

Figure S38. ESI mass spectrum of **5b.**

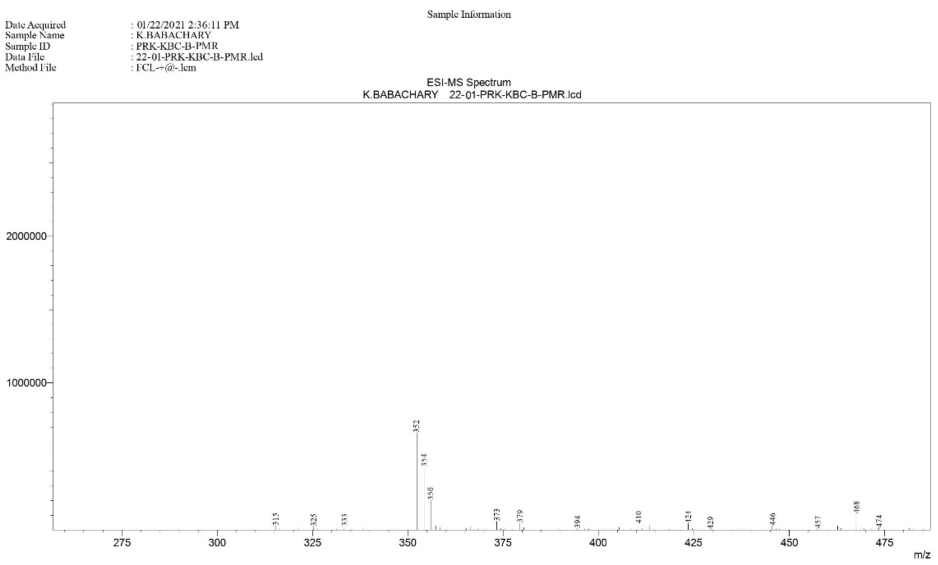

Figure S39. ESI mass spectrum of **5c.**

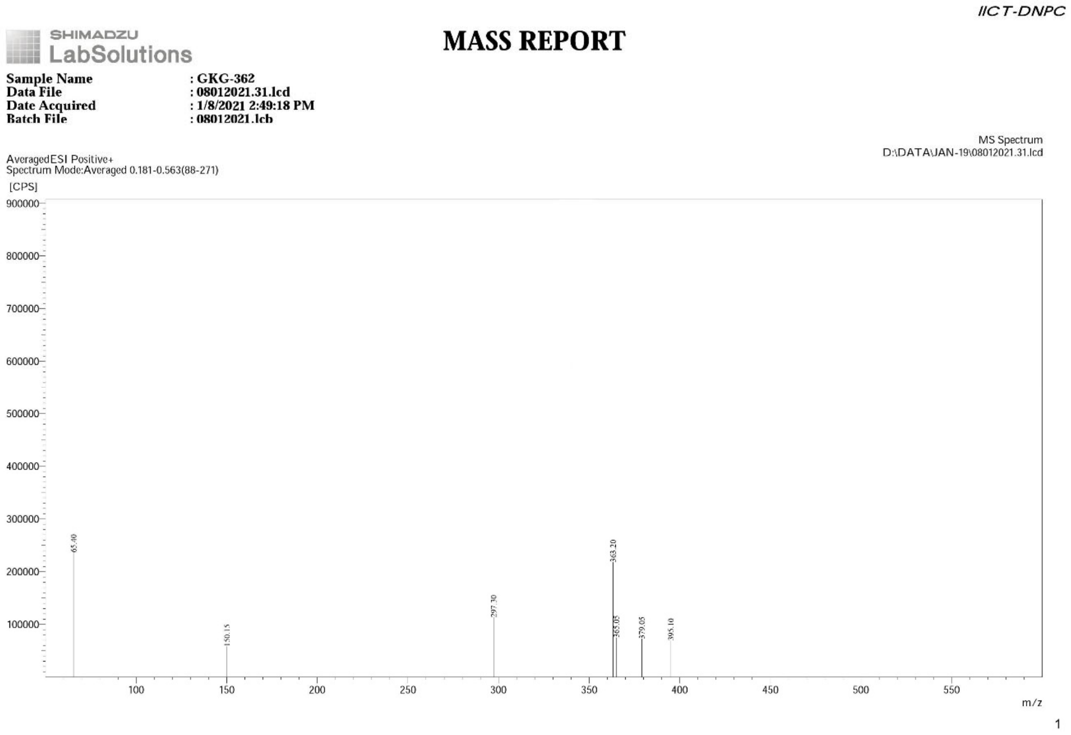

Figure S40. ESI mass spectrum of **5d.**

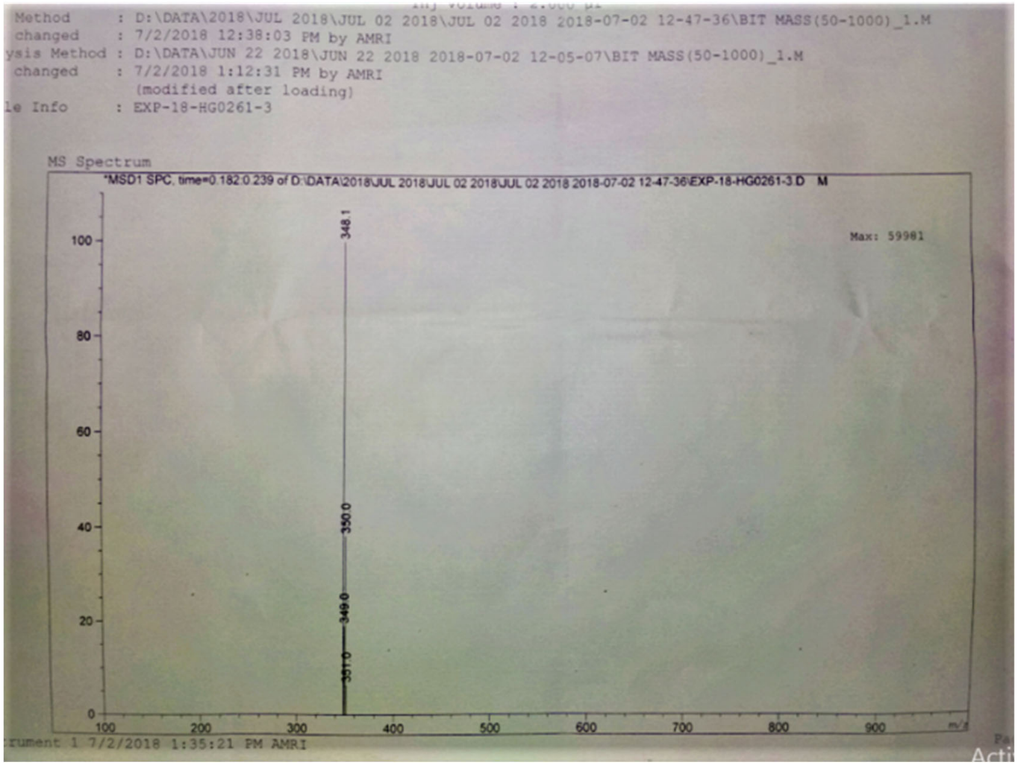

Figure S41. ESI mass spectrum of **5e.**

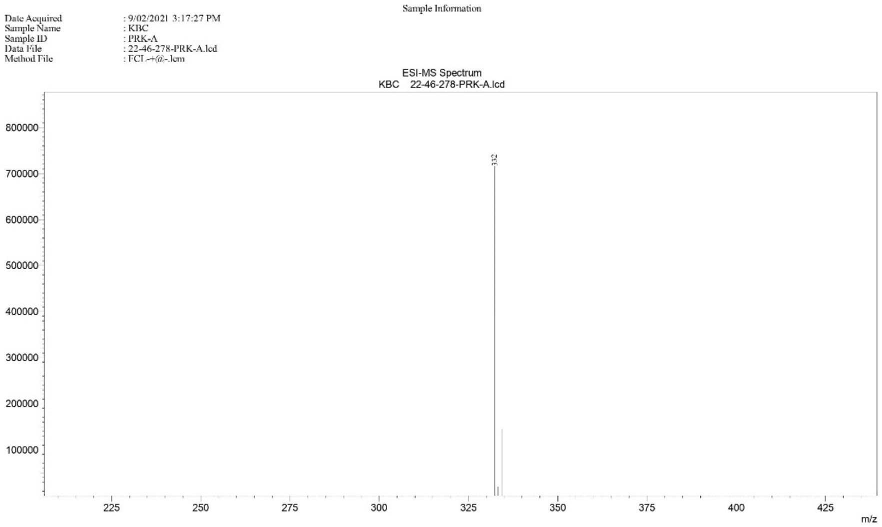

Figure S42. ESI mass spectrum of **5f.**

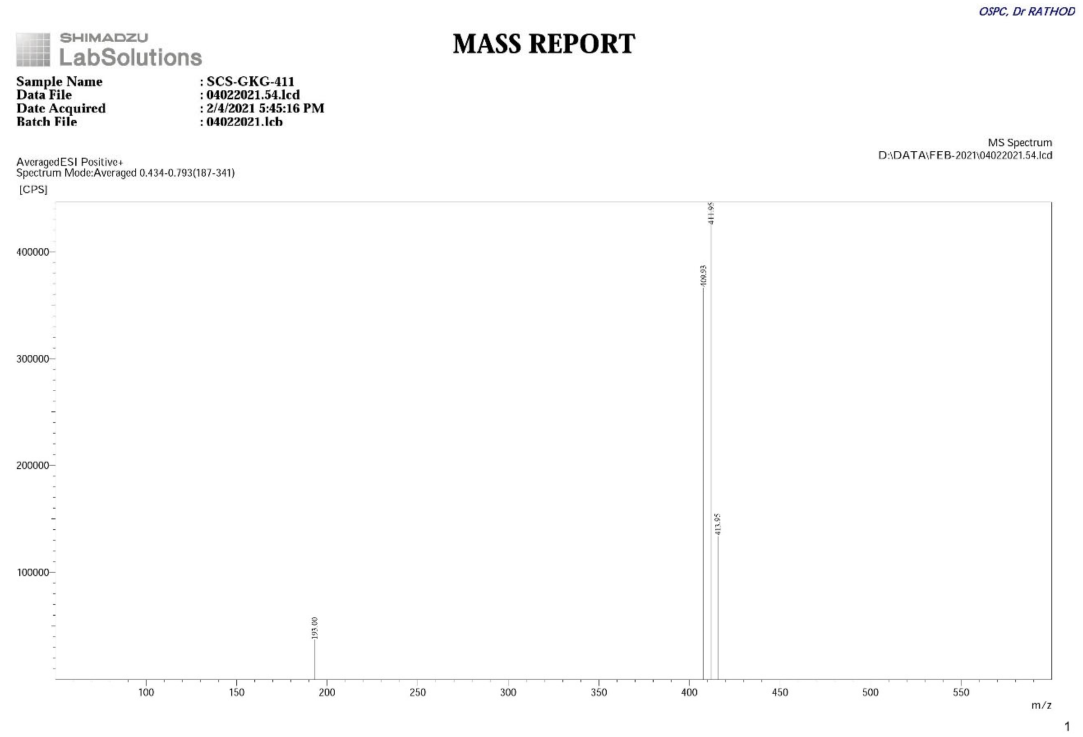

Figure S43. ESI mass spectrum of **5g.**

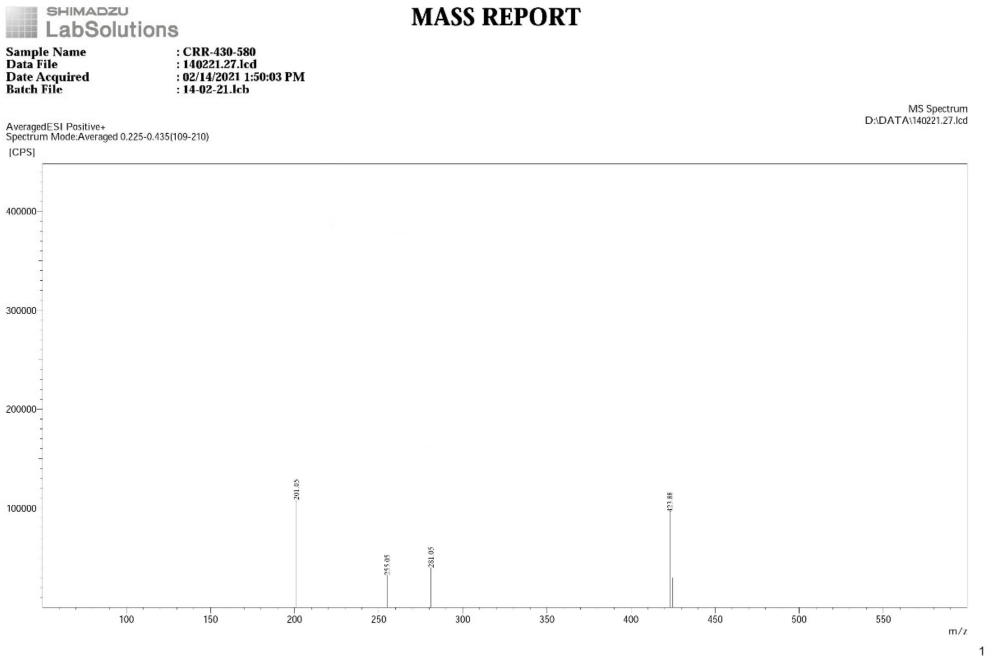

Figure S44. ESI mass spectrum of **5j.**

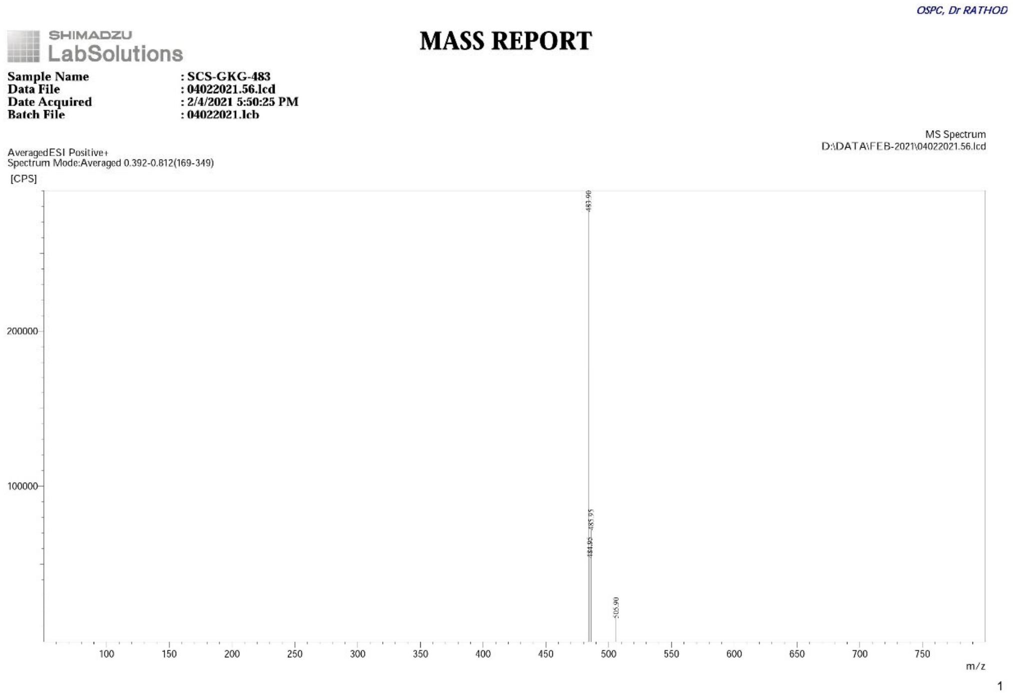

Figure S45. ESI mass spectrum of **5l.**

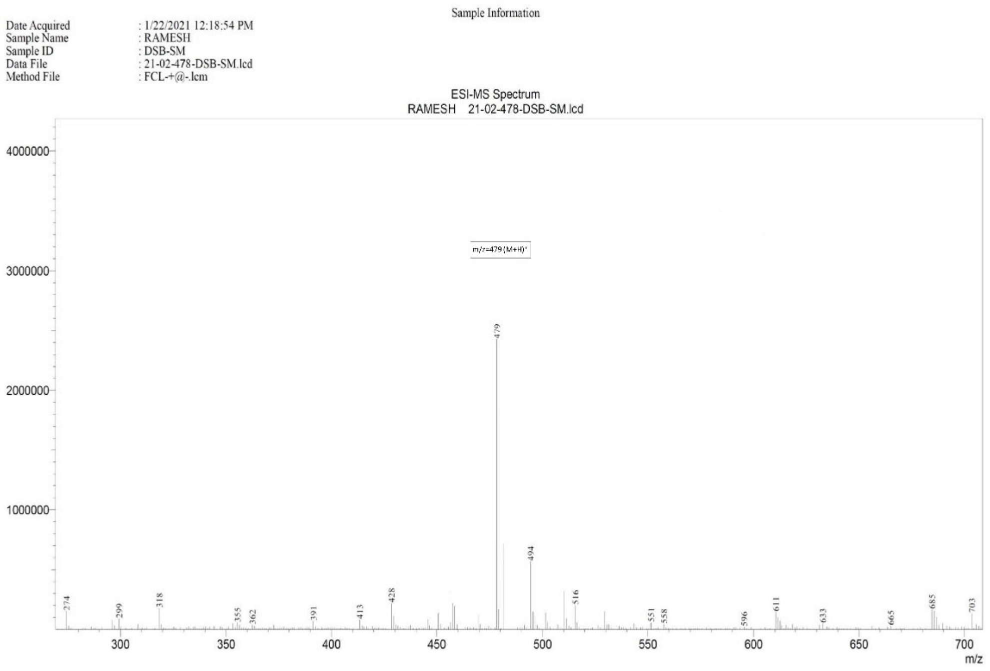

Figure S46. ESI mass spectrum of **5n.**

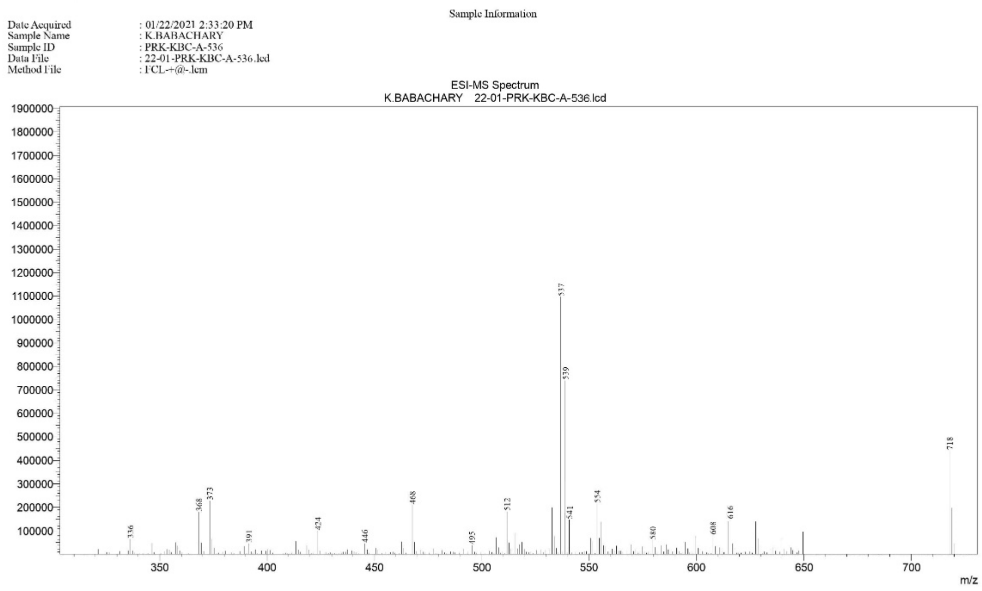

Figure S47. ESI mass spectrum of **5q.**

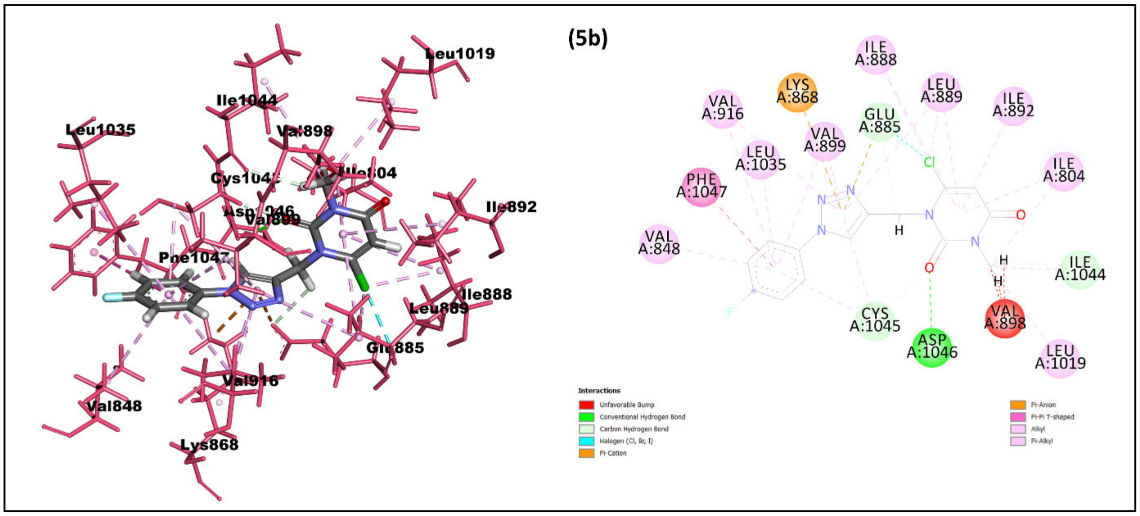

Figure S48. Docking interactions of **5b** in the active site of the VEGFR-2 protein.

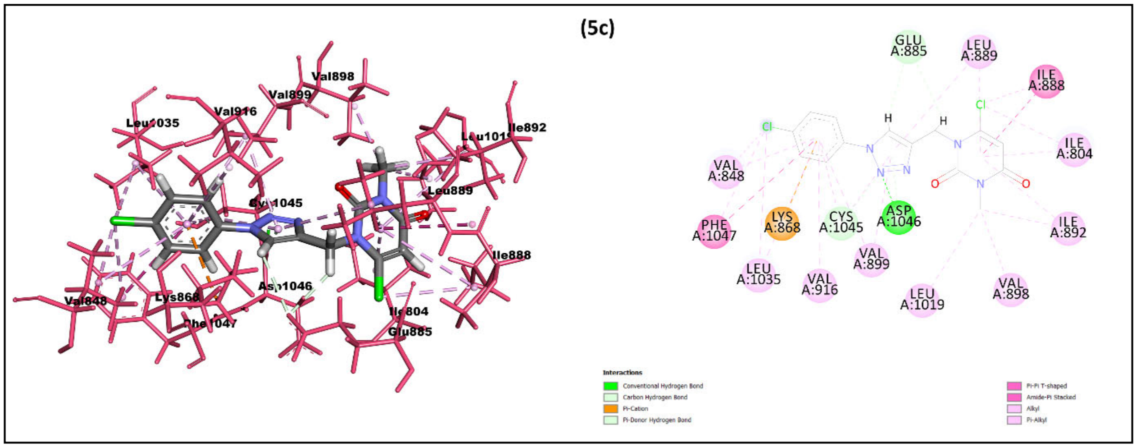

Figure S49. Docking interactions of **5c** in the active site of the VEGFR-2 protein.

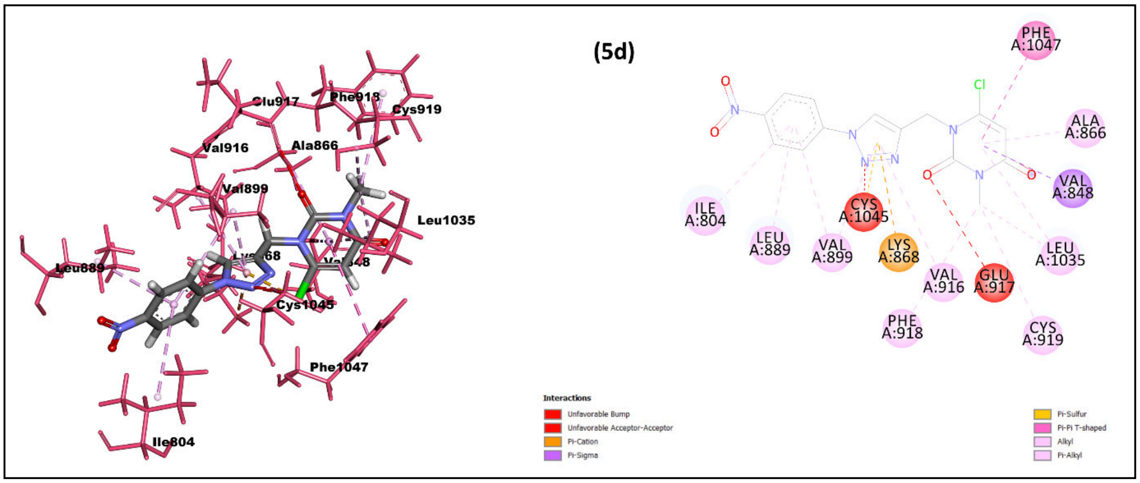

Figure S50. Docking interactions of **5d** in the active site of the VEGFR-2 protein.

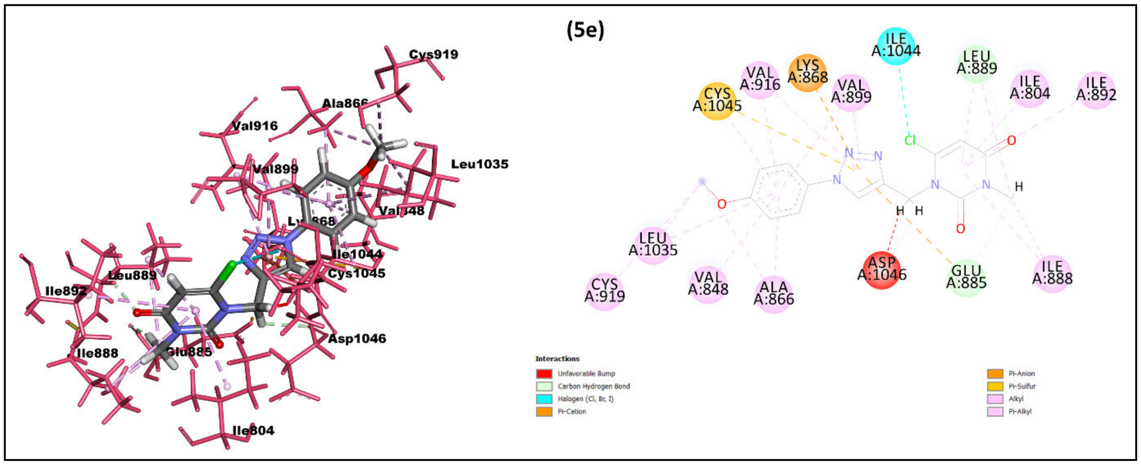

Figure S51. Docking interactions of **5e** in the active site of the VEGFR-2 protein.

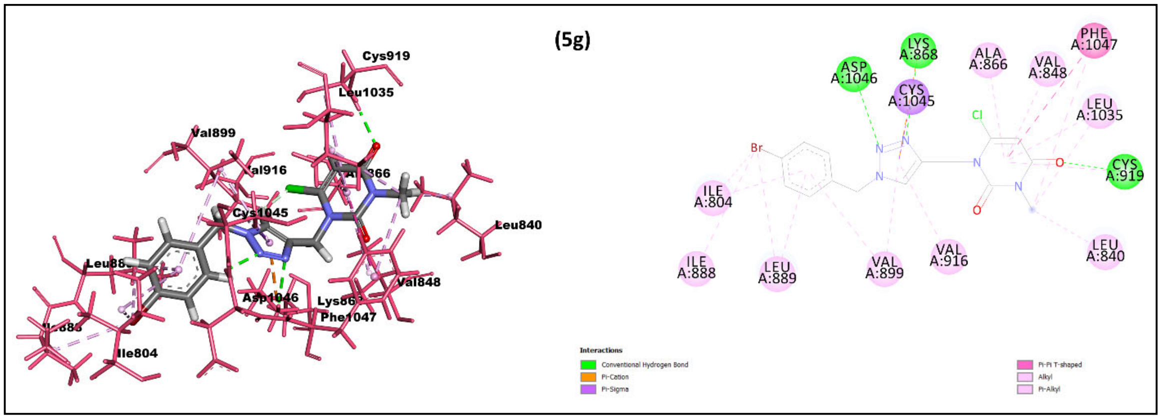

Figure S52. Docking interactions of **5g** in the active site of the VEGFR-2 protein.

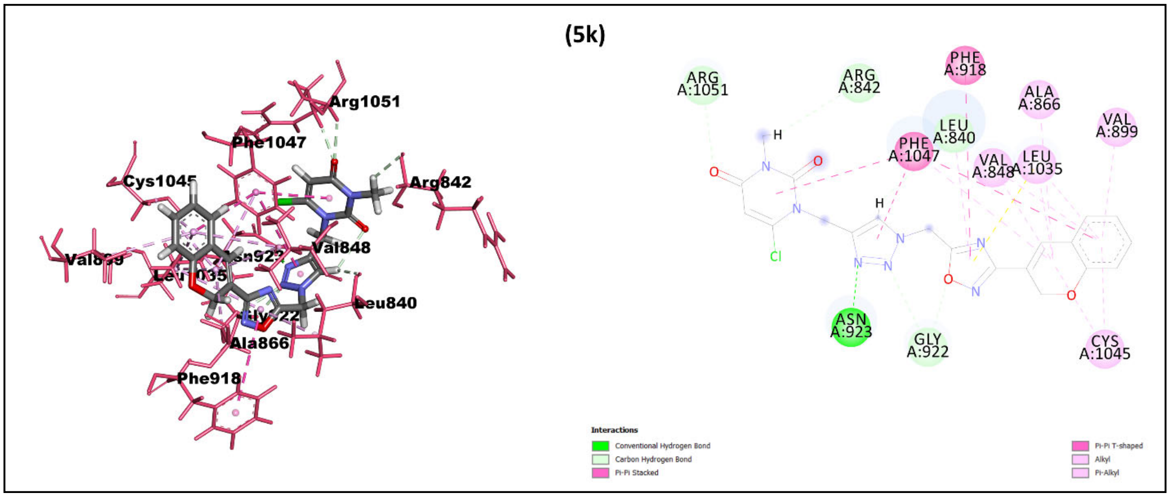

Figure S53. Docking interactions of **5k** in the active site of the VEGFR-2 protein.

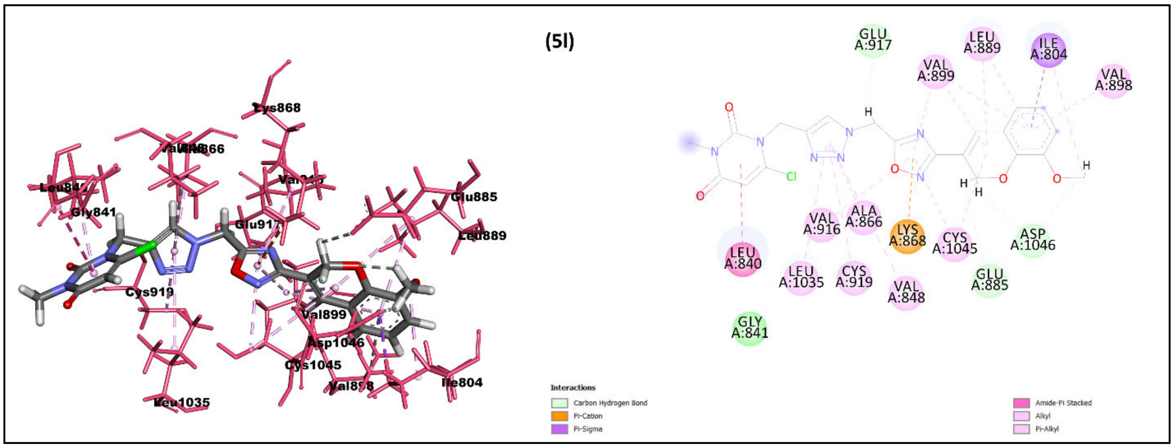

Figure S54. Docking interactions of **5l** in the active site of the VEGFR-2 protein.

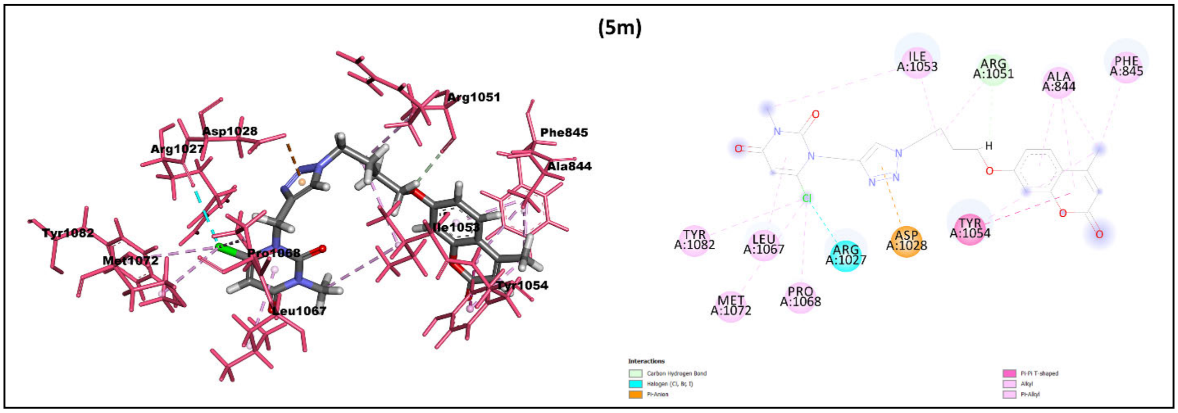

Figure S55. Docking interactions of **5m** in the active site of the VEGFR-2 protein.

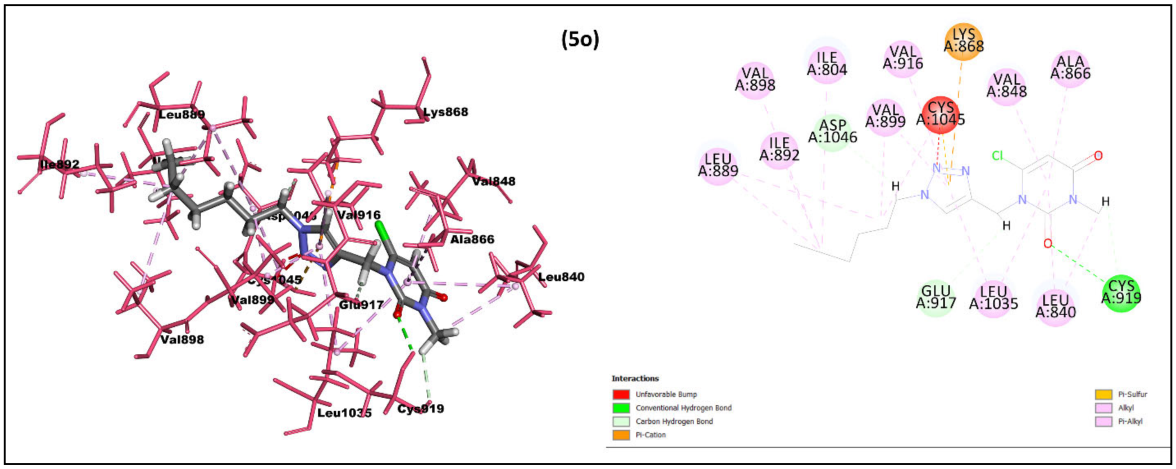

Figure S56. Docking interactions of **5o** in the active site of the VEGFR-2 protein.

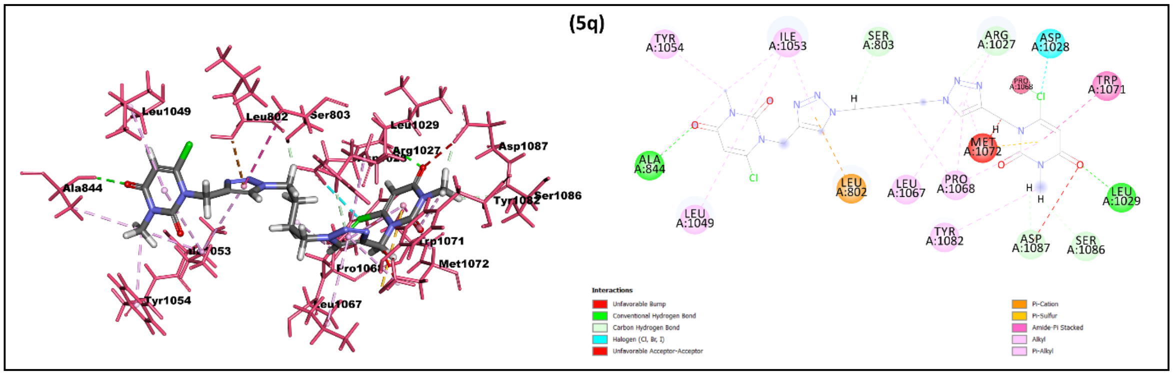

Figure S57. Docking interactions of **5q** in the active site of the VEGFR-2 protein.
